# Exchange and Communal Orientations (ECO) scale: The construction and validation of a method to measure target-specific relational orientations

**DOI:** 10.1371/journal.pone.0325232

**Published:** 2025-06-03

**Authors:** Agata Gasiorowska, Anna O. Kuzminska, Tomasz Zaleskiewicz

**Affiliations:** 1 Wroclaw Faculty of Psychology, SWPS University, Wroclaw, Poland; 2 Faculty of Management, University of Warsaw, Warsaw, Poland; Tianjin University, CHINA

## Abstract

This paper introduces the ECO Scale—a novel self-report measure to assess the degree to which individuals exhibit a dispositional tendency to orient themselves according to communal or exchange rules when establishing and maintaining relationships with others. In a series of six studies (total *N* = 3,252) conducted with diverse samples of Polish, American, and British participants, we documented that the 20-item ECO Scale in two separate language versions (English and Polish) is a psychometrically solid measure that can be used in different research settings. In Studies 1–3, we demonstrated the two-dimensional structure of the scale and provided support for configural, metric, residual, and partial scalar invariance of the scale across Poland and the United States (US). Study 2 supports the discriminant and construct validity of the scale, and demonstrates gender differences across orientations. Study 3 confirmed the convergent and divergent validity of the communal and exchange dimensions. Study 4 demonstrated the high test-retest reliability of the scale. Finally, Studies 5 and 6 verified the diagnostic and predictive validity of the ECO Scale. Study 5 showed that a high level of communal orientation predicts perceptions of the ambiguous social situation as more communal, while a high level of exchange orientation predicts perceptions of the ambiguous social situation as more exchange-based. Study 6 demonstrated that relational orientations measured with the ECO Scale predicts not only the declared willingness to provide assistance in a situation characterized by purely communal, purely exchange, or conflictual communal and exchange cues, but also actual behavior in these situations.

## Introduction

The theoretical idea we explore in the present paper is that people’s orientation toward forming communal versus exchange relationships might be considered a dispositional characteristic. However, we propose that this disposition, rather than being entirely generalized, is tied to the nature of a specific interpersonal relationship (e.g., whether it involves a close other or an acquaintance) and certain situational factors, shaping a person’s behaviors when building and maintaining relationships. In other words, we define this orientation as a target-specific relational orientation. More specifically, we assume that: (1) people differ in the extent to which they prefer communal or exchange norms/rules when engaging in specific relationships; (2) these differences are relatively stable over time; (3) they are linked to other individual characteristics; and (4) they can predict behavior in specific relational contexts.

A communal orientation reflects a willingness to provide non-contingent help and a desire to support the other person’s well-being. An exchange orientation focuses on personal benefits and the balance between investments and rewards. This distinction was initially proposed by Clark and Mills [1–3]; however, they considered both orientations as two general personality traits and measured them as if the expression of a particular orientation was independent of the relationship’s characteristics. As relationships with friends, family members, colleagues, and acquaintances differ, we propose that the exchange and communal orientations should also be measured in a relationship-specific manner.

In this paper, we present the results of a project aimed at developing and validating a new method for assessing individual differences in relational orientations defined as target-specific exchange and communal orientations. Next, we provide a detailed explanation of the differences between exchange and communal relationships, as well as individual differences in relational orientations. We then provide the rationale for developing a new scale. Finally, we report on the development and validation the Exchange and Communal Orientations Scale (ECO Scale).

### The psychology of communal and exchange relationships

The nature of communal relationships implies that benefits are given without the giver or receiver feeling obligated to reciprocate [2]. These benefits may take the form of goods, services, compliments, support, or symbols of care. Communal relationships are gratuitous and asymmetric, meaning that people offer benefits not because they expect something in return but because they seek to enhance the recipient’s happiness. The main motivation for providing benefits to another party is to express care, fulfill the recipient’s needs, and understand their perspective. People in a communal mode exhibit helpfulness, kindness generosity, and altruism [4]. In contrast, receiving a benefit in exchange relationships creates an obligation to return a similar one [2]. Each party in such a relationship primarily focuses on their own needs and closely monitors the ratio of costs and benefits. When one party provides something, they expect prompt repayment of comparable value [2,4].

Importantly, Clark and Mills [2] Relationship Theory differs from Social Exchange Theory [5] and the norm of reciprocity [6], despite their shared focus on interpersonal exchanges. Social Exchange Theory assumes that all relationships operate on a cost-benefit analysis, where individuals seek to maximize rewards and minimize losses. Similarly, the norm of reciprocity suggests that people feel obligated to return favors to maintain fairness and social balance. While Social Exchange Theory aligns with exchange relationships, it does not fully explain the selfless giving seen in communal bonds. Likewise, the reciprocity norm functions as a broad social rule but does not distinguish between relationships where reciprocity is obligatory and those where it is optional or delayed. Thus, Clark and Mill’s theory provides a more nuanced understanding of when and why people give in relationships, highlighting the distinction between calculated exchanges and care-driven interactions.

### Individual differences in relational orientations and their measurement

Clark and Mills [2] noted that exchange relationships are commonly found in business-type interactions or among acquaintances where there is no desire for friendship or intimacy. In contrast, communal relationships are typically exemplified by connections with friends, family members, or romantic partners [2]. Most people adhere to communal norms in prototypically communal relationships and follow exchange rules in transactional contexts. However, exchange norms can also be observed in close, intimate relationships [7,8], suggesting that some individuals apply exchange principles in typically communal interactions.

Clark et al. [1,2,9] proposed that the distinction between communal and exchange relationships may also be reflected in individual differences in relational orientations, shaping how people experience their interactions with others. According to this perspective, individuals differ in their tendency to follow communal and exchange-related norms in their relationships. These dispositional characteristics are referred to as “communal orientation” and “exchange orientation” [2]. In their theoretical model, Mills and Clark [9] even suggested that communal and exchange orientations function as personality traits and tended to treat them as such. In other words, these authors argued that while some individuals are primarily motivated by the balance of costs and benefits, others act out of concern for others.

Based on their theoretical considerations, Clark and Mills [2] developed two independent scales to measure general relational orientations: the Communal Orientation Scale (COS) and the Exchange Orientation Scale (EOS; see also [1,9]). The COS consists of items such as “When making a decision, I take other people’s needs and feelings into account.” In contrast, a sample item from EOS is: “When I give something to another person, I generally expect something in return.” The results of previous studies (see [2] for a review) have shown that the COS can predict various prosocial behaviors such as willingness to help others [1], willingness to express feelings to a relationship partner [10], equal distribution of goods [11], greater recognition of a partner for successful joint achievements [12], and volunteering in the form of political activism and civic engagement [13]. Low levels of communal orientation predicted burnout and depression in nurses and caregivers of Alzheimer’s patients [14,15] and abuse in close relationships [16]. Although research on the correlates of the EOS has been limited, findings suggest that an exchange orientation can predict retaliation [17] and the perception of romantic partners as means to personal goals [18].

The COS/EOS instruments assess participants’ communal and exchange orientations in general, without referencing specific individual relationships. However, Clark and Mills [2] noted that the same person may adopt a communal approach in some relationships while being exchange-oriented in others, suggesting that relationship orientation is target-specific. Consequently, because people’s orientations may vary across relationships, these two scales cannot accurately predict orientations in a given relationship, that is, they cannot capture a target-specific relationship orientation. Indeed, researchers have distinguished between general communal motivation toward others [1], often referred to as “communal orientation,” and partner-specific communal motivation, referred to as “communal strength” or “communal responsibility” [19–21]. Communal strength, as assessed by scale developed by Mills et al. [21], refers to how much a person feels responsible for the well-being of a particular communal partner. It can also be characterized as the willingness to bear certain costs to help someone in need, the emotional distress one would experience if unable to fulfill obligations to a communal partner, or the guilt resulting from neglecting the partner’s needs [21]. The 10-item questionnaire can be completed for different individuals, such as best friends, immediate family members, or acquaintances [21].

At the same time, no fully validated scales exist in the literature for assessing target-specific exchange orientation. This may be because, as Clark and Mills [2] noted, while the same person can establish relationships of varying strength with different communal partners, there is no equivalent concept of “exchange strength”—all exchange relationships are similar. Nonetheless, in this project, we assumed that the extent to which individuals follow exchange norms in a given relationship may depend not only on personal characteristics but also on the nature of the relationship. Interestingly, although Clark and Mills [2] identified relationships between acquaintances, strangers meeting for the first time, and business partners as prototypical examples of exchange relationships, they also provided an example of someone who perceives their relationships with relatives in a similar way. Moreover, as we have noted, some individuals—presumably those with high levels of general exchange orientation—are more likely to apply exchange norms even in close, intimate relationships [7,8]. Indeed, the prevalence of market values in modern Western societies suggests that individuals may be increasingly inclined to perceive and interpret reality through the lens of exchange relationships [22]. Consequently, they might conclude that a given relationship is transactional, which leads them to apply exchange-specific regulations and coordination mechanisms [23,24]. Therefore, we identified a clear need for a measure that goes beyond capturing a general level of exchange orientation and instead allows for assessing the propensity to apply exchange norms and rules within a specific relationship.

Some researchers have also recognized this need and attempted to adapt the instructions or items in general measures to refer specifically to the participant’s spouse or romantic partner. They reasoned that using a more generalized (i.e., original) scale would not adequately capture the dynamics of exchanges and communal orientations within close relationships [17,18,25]. However, such an approach could only be useful if the modified version of the scale is properly validated. Adapting the instructions and items of a scale may change its psychometric properties. Therefore, researchers should be cautious before applying such modifications without thoroughly testing the dimensionality, reliability, and validity of the adapted scale [26,27]. However, this was not the case in the aforementioned studies [17,18]. Therefore, following Furr’s [28] advice that “well-validated, original scales are preferable to modified scales” (p. 9), we decided to develop and validate a new scale based on the theory of Clark and Mills [2]. The aim of the scale is not only to measure exchange and communal relationship orientations but also to consider the specific target or nature of the relationship.

From the theoretical perspective, a target-specific measure provides a nuanced understanding of how individuals adapt their relational strategies based on the unique dynamics of a particular relationship. While trait-level orientations provide a broad perspective, they cannot account for variations in behavior that result from different relational contexts. For example, a person may adopt a communal orientation toward one colleague while maintaining an exchange orientation toward another. Recognizing such specificity can enrich our understanding of the mechanisms underlying relational behavior and provide a more granular framework for analyzing interpersonal dynamics.

In practice, the proposed measure could improve the predictive accuracy of relational outcomes. For example, in an organizational context, knowing whether an employee demonstrates a communal or exchange orientation toward his or her supervisor and how that fits the supervisor’s expectations could predict job satisfaction, trust, or performance better than a general trait measure. In clinical or counseling contexts, a targeted approach could help identify relational incongruence or conflict between spouses in their relational orientations and provide insight to make consequent interventions more effective. Therefore, we felt that developing a valid scale to measure goal-specific communal orientation (communal strength) and exchange orientation in a comparable manner would significantly enhance the ability to test predictions about behaviors characterized by varying degrees of relationship orientations.

### Measuring exchange and communal orientations: scale blueprint and overview of the present research

The main goal of our project was to develop a new method for measuring exchange and communal orientations that would be free from the limitations discussed in the previous section. In developing this method, we followed several principles. First, we created a scale with dimensions that identify exchange and communal orientations as defined by Clark and Mills [1,2,9]. The target-specific communal orientation was defined as the tendency to offer help unconditionally and the desire to promote the welfare of a specific partner. Conversely, the target-specific exchange orientation referred to the expectation of an equal relation between costs and benefits, and the tendency to prioritize personal gain when interacting with a particular partner (see Table 1). In our theoretical model, we assumed that exchange and communal orientations toward a specific other are two distinct but related dimensions. It means that individuals can simultaneously have high or low scores on each dimension, indicating their willingness or reluctance to apply certain norms to their relationship with a particular partner. Although we believe this is relatively rare, some individuals may score high on both exchange and communal orientation toward a specific partner, depending on the interactional context (e.g., relationships with a colleague at work vs. in a private setting). It is also conceivable that someone could score low on both dimensions, indicating a lack of interest in building a relationship with the target.

**Table 1:** Blueprint for the ECO scale.

Area of content	Exchange orientation	Communal orientation
Motivation to provide benefits to the other party	Focusing on own needs. Expecting something in return. Balance in giving and receiving benefits. Preferred benefits are specific and measurable.	Focusing on the needs of the other party. Showing concern, meeting one’s needs, and taking a perspective. Non-specific, hardly measurable benefits predominate (e.g., support, and closeness as motive for transferring benefits, even in money).
Payment/compensation	Expected immediate payment (at a given time). Preference for proportional compensation. Equal exchange. Each one gets according to their merit.	Compensation/payment is undesirable. Preference for deferred (undefined) reciprocity. No expectation of proportionality. Asymmetric exchange. Each one receives according to their needs.
Shared tasks and resources	Input and profit tracking (merit-based). Tracking of one’s own needs. Taking time to think about equity/balance.	No tracking of inputs and profits. Tracking of other people’s needs. Taking time to think about the other party of the relationship.
Helping	Lack of spontaneous help to others. Not asking for help. Spontaneous, disinterested help is not expected. Refusing help is not problematic.	Spontaneous help for others. Asking for help. Spontaneous, disinterested help is expected. Refusing help leads to a deterioration of mood.
The strength of a bond and the will to stay connected	Meager bonding strength. Willingness to maintain contact varies depending on the context and is present only in an exchange/self-interest context.	The strength of ties and willingness to maintain contact varies depending on the situation (weak or strong community ties).

Second, we aimed to develop a scale with instructions allowing researchers to specify a particular target person against whom the relational orientations would be measured. Third, we wanted to develop a method that would be available in parallel in two languages—English and Polish—to demonstrate the cultural invariance of the measurement. Fourth, we aimed to validate our method comprehensively, encompassing various aspects of reliability and validity, including convergent and discriminant validity.

In the remainder of this article, we focus on the development and validation of the ECO scale in both Polish and English. We conducted six preregistered studies in three countries, in which we created two language versions of the measure (English and Polish), validated them, and established a nomological network of the scale. In Study 1, we demonstrated the process of creating the ECO scale, verified its two-dimensional structure, and provided preliminary evidence for the configural, metric, and partial scalar invariance of the scale across the two countries (Poland and the United States; see Table 2 for demographic data in all studies). Study 2 further confirmed the two-dimensional structure of the ECO Scale, in which exchange orientation and communal orientation function as two distinct but correlated constructs, provided initial evidence for the discriminant and construct validity of the scale, and showed gender differences in the level of exchange and communal orientations. Study 3 reconfirmed the two-dimensional structure of the ECO scale and its measurement invariance in samples from Poland and the USA. It also provided further evidence for the convergent and divergent validity of the two dimensions. Study 4 showed the high test-retest reliability of the scale. Finally, Studies 5 and 6 focused on testing the diagnostic and predictive validity of the ECO Scale. In Study 5, we showed that high levels of communal orientation predicted perceptions of an ambiguous social situation as more communal, whereas high levels of exchange orientation predicted perceptions of an ambiguous social situation as more exchange-based. These associations were also moderated by the degree of familiarity between the individuals to whom our experimental manipulation referred, demonstrating the specificity of relational orientation in relationships with a stranger and a close person. Finally, in Study 6, we found that relational orientations measured with the ECO scale predicted not only the declared willingness to help in a situation characterized by purely communal, purely exchange-related, or conflictual communal and exchange-related cues, but also actual behavior in these situations.

**Table 2:** Demographic details for studies 1–6.

Variable		Study 1		Study 2	Study 3		Study 4		Study 5		Study 6
Nationality		US	PL	US	US	PL	US	PL	US	PL	UK
*N*		441	458	413	416	438	223	268	296	300	1007
Gender	Women	48.53%	50.44%	47.97%	49.76%	50.00%	47.09%	51.87%	45.3%	46.3%	51.7%
	Men	49.89%	49.34%	50.60%	48.80%	49.09%	51.12%	48.13%	53.7%	53.4%	47.6%
	Not specified	1.59%	0.22%	2.42%	1.44%	0.91%	1.79%	0%	1.0%	0.3%	0.7%
Age	*M*	36.46	32.66	37.39	38.80	30.96	37.93	35.11	41.04	31.38	42.66
	Median	35	32	35	35	3	37	33	39	30	40
	*SD*	10.97	9.48	12.64	14.85	9.02	11.12	8.60	14.84	9.32	13.69
Ethnicity	Asian	6.28%	0%	9.83%	5.80%	0%	2.69%	3.73%	5.07%	0%	4.27%
	Black	4.99%	0.20%	5.01%	6.40%	0.20%	3.14%	4.85%	4.39%	0%	2.28%
	Mixed	5.55%	0.80%	4.43%	5.40%	0.60%	4.04%	2.24%	4.05%	0.32%	3.08%
	Other	1.66%	0.40%	2.31%	3.80%	0.80%	0.90%	1.12%	3.38%	0.97%	0.40%
	White	78.00%	96.22%	75.34%	76.80%	96.40%	86.55%	85.45%	77.03%	91.88%	79.34%
	Not specified	3.52%	2.38%	3.08%	1.80%	2.00%	2.69%	2.61%	6.08%	4.22%	10.63%
Income	*M*				3.46	4.34			3.48	4.14	
	Median				3	4			3	4	
	*SD*				2.93	2.67			2.93	2.48	
SES	*M*				4.93	5.52			4.98	5.24	
	Median				5	5			5	5	
	*SD*				1.88	1.50			1.87	1.48	

### Study 1

The aims of Study 1 were to reduce a large initial collection of items to only psychometrically sound items that would comprise the ECO scale in its Polish and English versions, to conduct an initial test of the factor structure of the scale, and to determine cross-cultural measurement invariance. We assumed that the ECO scale would encompass two different aspects of interpersonal relationships, namely exchange orientation (EO) and communal orientation (CO). In the instructions, we asked participants to refer to their behavior in a specific relationship and not their “average” or “general” behavior (see Supporting Information), and we allowed the description of such a person in the instructions to be changed or experimentally manipulated. Therefore, we developed the items so that they referred to a relationship with a specific person but, at the same time, were universal (relevant for different target persons/relationships). We aimed to avoid creating items that were too specific or unlikely to cause variability in responses. While we did not set detailed criteria for this definition, we carefully considered each item’s potential to elicit different responses, particularly when linked to a specific relational target. For example, we would consider the item “Our meetings are mainly driven by the need to pursue common interests” to be too specific, as it would be irrelevant to relations with a close person living in the same household. We would also consider an item like “Our bond is getting stronger and stronger” as unlikely to lead to variability in responses, as participants would answer “strongly disagree” when evaluating all target persons with whom they had not formed such a bond. We also ensured that the items were worded in simple language by keeping the sentences short, avoiding complex sentence structures, using familiar words, and limiting complex words so that people from different educational backgrounds could easily understand the scale. We assumed that participants would respond to items on a 5-point Likert scale, ranging from 1 = “strongly disagree” to 5 = “strongly agree.” On this basis, we created an initial pool of 45 items measuring EO, and 50 items measuring CO.

To assess the content validity of the scale and the simplicity of the language, 10 social psychologists (academics: eight women, two men, seven of whom had a doctorate in psychology, three were doctoral students in psychology, and all had experience in developing and validating psychological tests) were presented with the same definitions and conceptualizations of both orientations that we had used in creating the items (see Table 1) and asked to rate whether our items reflected either EO or CO on a scale from 1 = “This item does not meet the definition of [exchange/communal orientation]” to 5 = “This item perfectly meets the definition of [exchange/communal orientation]” (ICC = .65, 95% CI [.53,.74]). They also rated the comprehensibility of the items on a scale from 0 = “incomprehensible/ambiguous” to 5 = “simple and clear” (ICC = .69, 95% CI [.59,.77]). For each orientation, we then excluded items with average comprehensibility ratings of less than 4.5 out of 5 (17 for EO and 28 for CO). Finally, we selected the 20 items with the highest content validity ratings. The preliminary version of the scale consisted of 40 items (20 for each orientation). We then translated these items independently into English, reviewed the translations together, and produced a consensus version, which was then corrected by two native English speakers. Subsequently, two psychologists (academics) who did not know the original Polish items or the definitions of the underlying constructs independently translated the corrected version from English into Polish. Finally, all authors checked the back-translation against the original version and made the necessary corrections to both the English and Polish items to create the bilingual version of the scale.

Using the two pools of items, we collected data from the Polish and American samples. As our scale allows the specification of the particular target person of relational orientations, we chose an example of a close person and an acquaintance. We followed the idea of Clark and Mills [2] that relationships between acquaintances often represent exchange relationships, whereas communal relationships are often typical of relationships with friends, family members, romantic partners, or spouses. In this study, we aimed to measure exchange and communal orientations by using the target individuals with whom people prototypically form communal and exchange relationships. Therefore, we asked each participant to complete the ECO scale questionnaire twice (with respect to a close person and a mere acquaintance).

In Study 1, we also examined the dimensionality of relational orientations. The authors of some previous works, including Bresnahan et al. [29] and Buunk et al. [30], found no empirical support for the existence of distinct communal and exchange orientations, suggesting that these orientations are not independent but instead represent opposite ends of the same underlying dimension. In other words, a low level of communal orientation towards a specific partner may indicate a high level of exchange orientation in that particular relationship. This assumption can also be observed in studies where relationship norms have been manipulated: These studies show that behaving according to communal principles weakens adherence to exchange-based rules, and vice versa [31–33]. In our theoretical model, however, we assumed that exchange and communal orientations toward a specific person are correlated but distinct constructs. Therefore, to examine the structure of communal and exchange orientations in a relationship with a given individual, we tested two competing models of the ECO scale: (1) a model with EO and CO as two ends of the same dimension, and (2) a model with EO and CO as two separate but correlated dimensions. We demonstrated that the latter model fit the data better, so we only used this model for further analyses. We selected the items for the final version of the ECO scale and retested its factor structure.

Finally, in Study 1 we collected initial evidence for cross-cultural measurement invariance. We tested measurement invariance to ensure that a measurement model is equivalent across countries and languages, allowing researchers to confidently compare latent constructs without bias, and ensuring that observed differences are meaningful and not due to measurement discrepancies. Configural invariance ensures that the basic structure of a measurement model (e.g., the number of factors and the pattern of factor loadings) is the same across groups or time points. This is the foundational step, confirming that the measured construct is conceptually similar across contexts. Metric invariance (also called weak invariance) tests whether the factor loadings are equal across groups or time points. This ensures that the strength of the relationship between observed variables and their underlying latent constructs is comparable, so that meaningful comparisons of the relationships between the variables are possible. Finally, scalar invariance (strong invariance) examines whether the intercepts of the observed variables are equal across groups or time points. Scalar invariance is essential for comparing the means for latent factor, as it ensures that the observed differences in means are not due to systematic measurement errors but reflect actual differences in the underlying construct [34,35].

The study hypotheses, design, sample size, and analyses were preregistered at https://aspredicted.org/txk4-br9y.pdf. The full wording of the scale in its Polish and English versions are provided in Supporting Information.

## Materials and methods

This and the following studies were performed in accordance with the Declaration of Helsinki and were registered and approved by the Ethical Committee of the SWPS University, decision number 07/P/04/2020. All participants in all studies provided informed consent by clicking within the survey system. We reported how we determined our sample size, all data exclusions (if any), all manipulations, and all measures in the study, and we followed JARS [36]. All data and materials are available at https://osf.io/76pk2/. All studies were preregistered.

In Study 1, we recruited 1,047 participants from Prolific Academic to participate in a study in exchange for £1 (*n* = 541 from the US; *n* = 506 from Poland). Data collection lasted from January 31, 2022, to February 2, 2022. We excluded 100 participants from the US and 48 participants from Poland either because they did not provide valid responses to one or more attention checks or they did not provide a description of their assigned person. The first attention check was the open-ended question in the demographics section: “Please provide the current date.” The other two attention checks were questions embedded in the scale: “Please choose ‘Strongly agree’ in response to this item” and “Please choose ‘Strongly disagree’ in response to this item.” The final sample consisted of 899 participants (see Table 2 for demographic details).

After participants gave their informed consent, they were asked to think of two people in random order: (1) an adult person who was important to them, and with whom they had a close relationship (friend, life partner, or adult child), and (2) an adult person they had only recently me, and did not yet know whether this relationship would develop in any way (i.e., this person could be a potential new friend or someone who would soon fade from their memory). After giving a brief description of the person (along with the name of a close person), the participants were asked to respond to 40 items that measured communal and exchange orientations. The order of the items relating to each person was randomized.

## Results

### Confirmatory factor analysis and item selection

As a first step, we tested the two competing models in confirmatory factor analysis (CFA) using pooled Polish and American samples: (1) a model with EO and CO as two ends of the same dimension, and (2) a model with EO and CO as separate but correlated dimensions. We included in each model separate latent variables, representing the evaluation of the relationship with the close person and with the acquaintance, and assumed that the latent variables were correlated. We performed CFA with the maximum likelihood estimation method and robust estimation of the standard errors—a procedure that accounts for the nonnormality of variables.

The first model, with two latent variables representing the dimensions of exchange versus communal orientation separately for the relationship with a close person and with an acquaintance, showed an acceptable fit to the data in terms of RMSEA = .072, 90% CI [.071,.073] and GFI = .96, but not when considering other indices, CFI = 0.62, TLI = 0.61, SRMR = .094. We observed significant and positive standardized factor loadings for the items measuring exchange orientation and significant negative factor loadings for the items designed to measure communal orientation (all *P*s* *< .001). Eighteen items, however, had factor loadings of less than .4. The correlation between the exchange (vs. communal) orientation in a close relationship and a relationship with an acquaintance was positive but weak, *r* = .16, *P *< .001.

The second model, with four latent variables representing the dimensions of exchange and communal orientation separately for the relationship with a close person and with an acquaintance, showed an acceptable fit to the data, with RMSEA = .057, 90% CI [.056,.058], SRMR = .077, and GFI = .92, but showed a lack of fit given the other fit indices, CFI = 0.76, TLI = 0.76. However, the overall fit of the model was better than the fit of the first model. All standardized factor loadings were significant and positive (all *P*s* *< .001), and only six were less than .4 (see S1 Table in Supporting Information). We found negative correlations between exchange orientation and communal orientation for both a close relationship, *r* = −.59, and for a relationship with an acquaintance, *r* = −.54. We also observed significant correlations between exchange orientations in the two types of relationship, *r* = .31, and between communal orientations in the two types of relationship, *r* = .13, all *P*s* *< .001.

To summarize, the second of our two preregistered models of scale structure, with separate EO and CO dimensions, appeared more promising for future research and was therefore used in further analysis. We analyzed the items with the highest factor loadings and the highest item-rest correlations separately for exchange and communal orientations, bearing in mind that we aimed to have the same set of items regardless of the type of relationship assessed (see S1 Table in the Supporting Information). However, the factor loadings and item-total correlations within the acquaintance and close person dimensions did not differ significantly for most items. If an item was considered conceptually redundant compared to others, we removed it, leaving the unique items that covered diverse aspects of our two constructs. In total, we identified 20 items, half of which measured exchange orientation and the other half communal orientation.

Using only these items, we tested the model with two latent variables representing the exchange versus communal orientation dimensions separately for the relationship with a close person and with an acquaintance. The model fit was below the acceptable thresholds for most indices, RMSEA = .102, 90% CI [.100,.104], GFI = .97, CFI = 0.63, TLI = 0.61, SRMR = .110. We then tested the second model, with four latent variables representing the dimensions of exchange and communal orientation separately for the relationship with a close person and with an acquaintance, using the same subset of items. The model fit the data better than the previous one, RMSEA = .068, 90% CI [.066,.070], SRMR = .065, GFI = .97, CFI = 0.84, TLI = 0.83. In this model, all standardized regression weights showed a moderate or strong relationship between the items and the latent factors (βs > .58, *P*s* *< .001; S2 Table in the Supporting Information). The correlations between the latent variables were similar to those observed in the two-dimensional model with all items, respectively *r* = −.51 for the correlations between exchange orientation and communal orientation in close relationships, *r* = −.48 for the correlations between exchange orientation and communal orientation in the relationship with an acquaintance, *r* = .36 for the correlations between exchange orientations in the two types of relationship, and *r* = .14 for the correlations between communal orientations in the two types of relationship, all *P*s* *< .001. In summary, the analyses conducted thus far allowed us to reduce a larger item pool to a 20-item scale and provided additional support for the two-dimensional structure of the ECO scale.

### Measurement invariance

In a second step, and in line with the preregistration, we conducted a measurement invariance analysis, using multigroup CFA [37] to assess the psychometric equivalence of the ECO scale across country groups. First, we evaluated the model with four latent variables separately for participants from Poland (*N* = 456) and from the USA (*N* = 441). We then examined the psychometric equivalence of the ECO scale across the two groups, testing configural, metric, and scalar invariance. Configural invariance ensures that the same factor structure is present in all groups; i.e., the number of factors and pattern of factor loadings are the same in all groups, but the actual values of the loadings may differ. Metric invariance, also known as weak invariance, builds on configural invariance by additionally assuming that the factor loadings are the same in all groups. This means that the relationship between the items and the latent variables is the same across groups, allowing for meaningful comparisons of the correlations between groups. Scalar invariance, or strong invariance, further extends metric invariance by assuming that the intercepts of the items are also the same in all groups. This means that any differences in the observed scores can be attributed to differences in the latent variables rather than differences in the measurement process [34,35], hence allowing for meaningful comparisons of the constructs’ mean values between groups.

We tested invariance according to model fit and change in fit indices (i.e., ΔRMSEA, ΔCFI, and ΔTLI). Following Cheung and Rensvold [35] and Vandenberg and Lance [34], we assumed that a change in RMSEA of .015 or less and a change in CFI and TLI of 0.01 or less would mean that the two models did not differ; between 0.01 and 0.02, that the two models might have possibly differed; and greater than 0.02, that the two models definitely differed.

For the initial test of the model with four latent variables separately for participants from Poland and the USA, the maximum likelihood CFA for the American sample yielded an acceptable model fit in terms of most but not all indices, RMSEA = .068, 90% CI [.065,.072], GFI = .96, CFI = 0.85, TLI = 0.84, SRMR = .069, which was similar to the CFA for the Polish sample, RMSEA = .069, 90% CI [.066,.073], GFI = .97, CFI = 0.82, TLI = 0.81, SRMR = .076, providing initial support for configural invariance. We, therefore, conducted a formal test of measurement invariance between participant groups in the following phases: (1) configural invariance, assuming the same factor structure in the two groups; (2) metric invariance—assuming configural invariance and additionally equal factor loadings from items to first-order factors and from first- to second-order factors); (3) scalar invariance, assuming metric invariance and additionally equal intercepts for items (Table 3).

**Table 3:** Measurement invariance across country groups in study 1.

Type of invariance	RMSEA	GFI	CFI	TLI	ΔRMSEA	ΔCFI	ΔTLI
Unconstrained model	.068	.965	0.837	0.827	–	–	–
Configural	.069	.963	0.837	0.827	.001	<.001	<.001
Metric	.069	.961	0.833	0.827	<.001	−.004	<.001
Scalar	.074	.958	0.802	0.801	<.005	−.031	−.026
Partial scalar	.070	.960	0.823	0.821	<.001	−.010	−.006

In terms of configural and metric invariance, ΔRMSEA, ΔCFI, and ΔTLI were below .01, suggesting that the structure of the scale and the factor loadings of the latent variables on the items did not differ between country groups. However, we found no support for scalar invariance when we imposed constraints on the item intercepts, implying that at least some item intercepts differed between the two countries. We further investigated whether partial scalar invariance could be achieved by using a backward approach and removing constraints on consecutive items. We found that removing restrictions on four items of the latent variable representing communal orientation in the relationship with an acquaintance and on four items of the latent variable representing exchange orientation in the relationship with a close person (see Table S2 in the Supporting Information) resulted in partial scalar invariance in terms of ΔRMSEA, ΔCFI, and ΔTFI.

Overall, we found that the measurement model was invariant across country groups with respect to CO in a relationship with a close person and EO in a relationship with an acquaintance. It is therefore possible to make cross-country comparisons, although the results regarding the dimensions where we only achieved partial scalar invariance should be treated with caution.

### Comparisons for a target person: a close person versus an acquaintance

In the final step, we averaged the scores for each of the dimensions of the ECO scale for a close relationship and a relationship with an acquaintance and conducted an additional, preregistered analysis comparing the scores for a close person and an acquaintance with two repeated-measures ANOVAs. As expected, the score on the communal orientation dimension was higher for the relationship with a close person (M = 4.41, SD = 0.65) than for the relationship with an acquaintance (M = 2.81, SD = 0.75), F(1, 898) = 2,714.42, P < .001, η^2^ = .751, and the score on the exchange orientation dimension showed the opposite pattern, namely M = 2.47, SD = 0.78 for the relationship with a close person and M = 3.35, SD = 0.71 for the relationship with an acquaintance, F(1, 898) = 945.98, P < .001, η^2 ^= .531. This pattern of results provided initial confirmation of the validity of the ECO scale and its dimensions.

## Discussion

Based on the results of Study 1, we selected 20 psychometrically sound items simultaneously in English and Polish, half of which were designed to measure exchange orientation and the other half communal orientation. The CFA supported the structure of this scale, which is based on different dimensions for exchange and communal orientations. We were also able to provide initial support for the measurement invariance of the scale in the two countries (Poland and USA). In terms of scalar invariance, we found that the measurement model of both countries was invariant with respect to communal orientation in the relationship with a close person and exchange orientation in the relationship with an acquaintance. However, we found evidence of only partial scalar invariance with respect to communal orientation in the relationship with an acquaintance and exchange orientation in the relationship with a close person. Thus, although we can compare the strength of correlations for relationship orientation and other constructs, cross-national comparisons for the mean scores of the dimensions should be treated with caution. Finally, as evidence for the validity of the ECO scale, we found that the score for the communal orientation dimension was higher in the relationship with a close person than in the relationship with an acquaintance, while the score for the exchange orientation dimension showed the opposite pattern.

### Study 2

In Study 1, we reduced a large pool of items to a 20-item scale in Polish and English and found initial support for its two-dimensional structure. The aim of consecutive Study 2 was to (1) confirm the adequacy of the ECO Scale factor structure in another sample; (2) investigate gender differences in communal and exchange orientation; and, most importantly, (3) test the convergent validity of the two dimensions of relational orientation [38]. To this end, a large sample of participants from the USA completed the ECO Scale with respect to a close person and an acquaintance, along with several other scales measuring constructs that we hypothesized to be related to either exchange orientation or communal orientation.

First, we expected that exchange orientation toward an acquaintance and a close person (as measured with the ECO scale) would be positively related to, but distinct from, general exchange orientation. At the same time, we expected that communal orientation toward both an acquaintance and a close person would be positively correlated with, but distinct from, general communal orientation as measured by Clark and Mills’s scale [2]. We also hypothesized that target-specific exchange orientation would correlate negatively with general communal orientation and that target-specific communal orientation would correlate negatively with general exchange orientation.

Second, we hypothesized that exchange orientation would correlate positively with the calculative mindset, as measured by the Kim et al.’s scale [39]. Similarly, we expected communal orientation to correlate positively with social connectedness, as measured with the UBC Social Connection scale [40]. Again, we predicted negative cross-correlations, i.e., that target-specific exchange orientation would correlate negatively with social connectedness, and that target-specific communal orientation would negatively correlate with the calculative mindset. We based these hypotheses on the definitional understanding of what a communal and an exchange relationship is: a communal orientation reflects the tendency to offer unconditional help and a desire to support partners’ well-being and maintain closeness, whereas an exchange orientation expresses the focus on personal benefits and the balance between investments and rewards [41–43].

Finally, we hypothesized that women would report higher levels of communal orientation toward a close person and a stranger than men. We based this hypothesis on findings showing that the neural reward system is more sensitive to prosocial rewards in women than in men [44] and that women excel in communal and relational behaviors, including prosociality [45] and conflict resolution [46]. However, we had no specific expectations regarding gender differences on the exchange orientation dimension. The study hypotheses, design, sample size, and analyzes were preregistered at https://aspredicted.org/vc7q-862p.pdf.

## Materials and methods

As preregistered, we recruited 505 American participants from Prolific Academic for a payment of £1. The study was conducted on February 21, 2022. We excluded 92 participants who did not provide valid responses to one or more attention checks (the same as in Study 1) or a description of their assigned person. The final sample comprised 413 participants (see Table 2 for demographic details).

After participants gave their informed consent, they were asked to complete six questionnaires: (1) a 20-item version of the ECO scale, concerning the person with whom they had a close relationship; (2) a 20-item version of the ECO scale, concerning an adult person they had recently met and about whom they could not yet say whether or not a relationship would develop; (3) a 14-item scale measuring communal orientation [2]; (4) a nine-item scale measuring exchange orientation [2]; (5) the 10-item Calculative Mindset (CM) scale [39]; and (6) the 10-item UBC Social Connection scale [40]. The ECO items were answered on a scale ranging from 1 = “strongly disagree” to 5 = “strongly agree”; the Clark and Mills [2] questionnaires use a scale that ranges from 1 = “extremely uncharacteristic of me” to 5 = “extremely characteristic of me,” and the UBC and CM scales use a 7-point Likert scale from 1 = “strongly disagree” to 7 = “strongly agree.” The order of the scales and the order of the items within each scale were randomized (see Supplementary Information for detailed information on the scales used in this study).

## Results

### Confirmatory factor analysis

First, we retested the structure of the ECO scale—the two-dimensional model in which we assumed that EO and CO were separate but correlated dimensions of the perception of a close relationship and a relationship with an acquaintance. We expected that the two latent variables representing the evaluation of the relationship with a close person on the exchange and communal dimensions would correlate with each other, as would be the two latent variables for the relationship with an acquaintance. We also assumed that the latent variables representing an exchange or communal relationship with a close person would correlate only with the corresponding latent variables for the relationship with an acquaintance.

We conducted a CFA with the maximum likelihood estimation method and robust estimation of standard errors. The model with four latent variables representing the exchange dimension and communal dimensions separately for the relationship with a close person and the relationship with an acquaintance showed an acceptable fit to the data in light of some, but not all, indices: RMSEA = .072, 90% CI [.069,.075], SRMR = .072, GFI = .75, CFI = 0.85, TLI = 0.85. All standardized factor loadings were significant and positive, and higher than .5. We observed significant and negative correlations between exchange orientation and communal orientation for both a close relationship, *r* = −.33, *Z* = −5.42, *P* < .001, and a relationship with an acquaintance, *r* = −.45, *Z* = −7.05, *P* < .001. We also observed significant correlations between exchange orientations in two types of relationships, *r* = .36, *Z* = 5.88, *P* < .001, and between communal orientations in two types of relationships, *r* = .15, *Z* = 2.52, *P* = .012. Again, we compared the two-dimensional model with the one-dimensional model, assuming that exchange orientation and communal orientation in the relationship with an acquaintance and a close person represent two sides of the same construct. Model fit was worse than for the bi-dimensional model, RMSEA = .122, 90% CI [.118,.122], SRMR = .147, GFI = .41, CFI = 0.58, TLI = 0.56. These results supported our hypothesized two-dimensional model, wherein exchange and communal orientation are distinct but correlated constructs.

### Divergent and convergent validity

To test the discriminant and convergent validity of our scale, we conducted a series of preregistered CFAs separately for the exchange and communal dimensions of the ECO scale to examine whether relational orientations measured by this scale are related to, but separate from, general communal and exchange orientations measured with the Clark and Mills [2] scales, CM (calculative mindset), and social connectedness. For each of the external constructs, we tested whether a three-factor model (in which one factor represented exchange or communal orientation toward an acquaintance; the second factor, exchange or communal orientation toward a close person; and the third factor, the related construct) fit the data better than a two-factor model (in which one factor was manifested in items measuring either exchange or communal orientation toward an acquaintance and the items measuring the related construct, while the second factor was manifested in items measuring exchange or communal orientation toward a close person, and the items measuring the related construct). If exchange and communal orientations toward a close person and an acquaintance differed from the related constructs, the three-factor model would provide a better fit than the respective two-factor model.

All the CFAs we performed showed that the three-dimensional models fitted the data better than the corresponding two-dimensional models (see Table 4 for details). This suggests that exchange orientation and communal orientation toward specific individuals—as we operationalized them—are distinct from communal and exchange orientations understood as general traits, CM, and a sense of social connectedness. Although these constructs are distinct, we still expected that exchange orientation toward specific individuals would be positively correlated with CM and exchange orientation, but negatively correlated with general communal orientation and the sense of social connectedness. We also expected that the pattern of correlations would be reversed for a communal orientation toward specific individuals.

**Table 4:** Confirmatory factor analyses testing the divergent validity of the EO-CO scales in study 2.

ECO scales included in the model	Additional scale included in the model	Three-factor model					Two-factor model					Model comparison	
		RMSEA	SRMR	CFI	TLI	GFI	RMSEA	SRMR	CFI	TLI	GFI	Δχ^2^ (2)	*P*
CO-C and CO-A	GCO	.083	.069	0.82	0.80	.96	.107	.104	0.69	0.67	.92	1015.74	***
	GEO	.075	.068	0.87	0.86	.98	.092	.087	0.80	0.78	.98	461.42	***
	CM	.068	.054	0.90	0.43	.97	.120	.144	0.68	0.66	.94	1616.54	***
	UBC	.077	.051	0.89	0.88	.96	.132	.169	0.68	0.66	.93	2067.58	***
EO-C and EO-A	GCO	.086	.083	0.77	0.75	.95	.115	.125	0.59	0.57	.96	1240.33	***
	GEO	.082	.062	0.82	0.80	.93	.091	.072	0.77	0.75	.92	253.64	***
	CM	.075	.059	0.86	0.85	.90	.111	.108	0.69	0.67	.79	1117.19	***
	UBC	.080	.054	0.87	0.86	.92	.161	.214	0.46	0.42	.85	3517.03	***

*Note*. *** *P* < .001

1. In the three-factor model, the first factor represents exchange/communal orientation toward an acquaintance, the second factor represents market/communal orientation toward a close person, and the third factor represents the related construct.
2. In the two-factor model, the first factor manifested in items measuring exchange/communal orientation toward an acquaintance and the items measuring the related construct. In contrast, the second factor manifested in items measuring market/communal orientation toward a close person and the items measuring the related construct.

EO-C: exchange orientation toward a close person; EO-A: exchange orientation toward an acquaintance; CO-C: communal orientation toward a close person; CO-A: communal orientation toward an acquaintance; GCO, GEO: general communal and exchange orientations measured with scales by Clark & Mills (2012); CM: calculative mindset measured by the Kim et al. [39] scale; UBC: social connection as a trait measured with a modified version of the UBC scale (Lok & Dunn, 2022).

We indeed found that most of the correlations we predicted were significant, with the exception of the correlations between exchange orientation (toward both a close person and an acquaintance) and social connectedness, and the correlations between communal orientation toward an acquaintance and CM (see Table 5).

**Table 5:** Correlations between variables measured in study 2.

Variable	*M*	*SD*	α	EO-C	EO-A	CO-C	CO-A	Fischer’s *Z*s (correlation comparisons)			
								EO-C vs. CO-C	EO-A vs. CO-A	EO-C vs. EO-A	CO-C vs. CO-A
EO-C	2.48	0.86	.95	–							
EO-A	3.31	0.81	.91	.32^***^	–						
CO-C	4.51	0.60	.90	−.29^***^	.06	–					
CO-A	2.91	0.76	.91	−.01	−.43^***^	.13^*^	–				
GEO	2.81	0.61	.73	.41^***^	.41^***^	−.12^*^	−.15^**^	8.00^***^	8.39^***^	0.12	0.51
CM	2.02	0.75	.90	.46^***^	.28^***^	−.17^***^	−.07	9.57^***^	5.13^***^	2.94^**^	1.50
GCO	3.64	0.60	.85	−.14^**^	−.14^**^	.32^***^	.28^***^	6.75^***^	6.15^***^	0.01	0.58
UBC	4.51	1.35	.95	−.04	−.05	.28^***^	.22^***^	4.70^***^	3.89^***^	0.14	0.95

*Note*. *** *P *< .001, ** *P *< .01, * *P *< .05.

EO-C: exchange orientation toward a close person; EO-A: exchange orientation toward an acquaintance; CO-C: communal orientation toward a close person; CO-A: communal orientation toward an acquaintance; GEO, GCO: general exchange and communal orientations measured with scales by Clark & Mills [2]; CM: calculative mindset measured with the Kim et al. [39] scale; UBC: social connection as a trait measured with the UBC scale [40].

We also conducted a preregistered analysis in which we compared the strength of the correlations between exchange and communal orientations toward a particular individual and the associated constructs. First, we found that the exchange orientation dimensions (toward an acquaintance and a close person) were more strongly correlated with CM and general exchange orientation than with social connectedness and general communal orientation. Second, we found that communal orientation (toward an acquaintance and a close person) correlated more strongly with general communal orientation and social connectedness than with CM and general exchange orientation (see Table 5). The strength of the correlations with related constructs for orientations toward an acquaintance and a close person was similar, with the only exception being the correlations between exchange orientation and CM. This correlation was significantly stronger for the orientation toward a close person than toward an acquaintance.

As the negative correlations between the target-specific exchange orientation and social connectedness were nonsignificant, same as the correlations between communal orientation toward an acquaintance and CM, we believed that these results further supported our assumption about the two relatively orthogonal dimensions of relational orientation. The very weak negative correlation suggested that, for example, a high score on the communal orientation scale is not simply the opposite of a CM. It rather suggests that communal orientation is independent of exchange orientation. Consequently, communal orientation cannot be equated with a low level of exchange orientation. This distinction underscores the idea that the relational dimensions we proposed are not mere opposites but represent two independent constructs. We therefore concluded that the pattern of the results supports the assumption that exchange and communal orientations towards a particular person are two distinct—albeit correlated—constructs.

### Gender differences in relational orientations

We conducted four ANOVAs in which compared the values for exchange and communal orientations toward a close person and an acquaintance between women and men. To reiterate, we expected that women would report higher levels of communal orientation toward a close person and toward an acquaintance than men. Still, we had no expectations regarding gender differences for the exchange orientation dimension. We found that women (*M* = 4.66, *SD* = 0.77) scored higher on communal orientation toward a close person than men (*M* = 4.38, *SD* = 0.64), *F*(1, 402) = 25.74, *P* < .001, η^2^ = .060. A similar, albeit weaker, pattern of results was found for a communal orientation toward an acquaintance, *M* = 3.02, *SD* = 0.75 for women, and *M* = 2.79, *SD* = 0.78 for men, *F*(1, 402) = 9.04, *P* = .003, η^2^ = .022. Men (*M* = 3.45, *SD* = 0.82) had higher scores on exchange orientation toward an acquaintance than women (*M* = 3.19, *SD *= 0.77), *F*(1, 402) = 9.86, *P* = .002, η^2^ = .024. Finally, we found no gender differences in exchange orientation toward a close person, *F*(1, 402) = 0.80, *P* = .370, η^2^ = .002.

## Discussion

In summary, the results of Study 2 further confirm the hypothesized two-dimensional structure of the ECO scale, in which exchange orientation and communal orientation are two distinct but correlated constructs. Furthermore, we demonstrated the construct validity of the scale by showing that exchange orientation and communal orientation toward specific individuals are related to, but distinct from, communal and exchange orientations understood as general traits, CM, and a sense of social connectedness. Finally, we found that women scored higher than men on communal orientation toward a close person and toward an acquaintance. However, men scored higher than women in the exchange orientation toward an acquaintance (but not toward a close person).

### Study 3

Having found initial support for the convergent and discriminant validity of the two dimensions of the ECO Scale, we had the following aims in Study 3: (1) to confirm the two-dimensional structure of the ECO Scale in different samples; (2) to test the measurement invariance of the final version of the ECO Scale; and (3) to further test the convergent and divergent validity of the two ECO Scale dimensions [38].

We recruited a large sample of participants from the US and Poland to complete the ECO Scale concerning a close person and an acquaintance, along with several scales measuring other constructs that, as we hypothesized, would be related to either exchange or communal orientations. This time, we expected that the exchange orientation as measured by the ECO Scale (toward an acquaintance and a close person) would be negatively related to other-interest, but positively to self-interest [47], materialism [48], and attachment-related insecurities (anxiety and avoidance) [49]. We expected a reverse pattern of correlations for communal orientation in the same relational contexts. This prediction was based on the theoretical distinction between communal and exchange orientations. First, individuals with a communal orientation prioritize the welfare of others and engage in need-based, altruistic behaviors, whereas those with an exchange orientation focus on self-interest and reciprocation-driven interactions [41–43]. Furthermore, communal orientation has been linked to less materialistic values, as it emphasizes intangible benefits like kindness and emotional support. In contrast, materialism aligns with the transactional focus of exchange orientation, where tangible and measurable outcomes are prioritized [50]. Finally, the relationship between relational orientations and attachment styles further supports our predictions. Secure attachment, which fosters positive relational attitudes and emotional stability, is associated with higher communal orientation. On the other hand, attachment insecurities, characterized by anxiety and avoidance, are associated with more negative relational attitudes and a diminished capacity for communal engagement [51]. Moreover, experimental studies have suggested that involvement in exchange or market-based relationships can act as a buffer against attachment insecurity, underlining its functional compatibility with exchange orientation [52,53].

We also expected that our relational dimensions would be linked to basic human values. More specifically, we predicted that exchange orientation measured with the ECO scale (toward both an acquaintance and a close person) would be negatively correlated with conservation and self-transcendence (social focus values [54]) and positively correlated with openness to change and self-enhancement (personal focus values [54]). In contrast, we expected that the correlations for the communal orientations would show the opposite pattern. We based these predictions on the observation that personal focus values—such as power, achievement, hedonism, and stimulation—emphasize personal success and the pursuit of dominance over others. Research shows that such values are more prevalent in contexts emphasizing individualism and economic development [55]. In contrast, social values include universalism, benevolence, conformity, and tradition, which prioritize the well-being of others and the environment. Studies show that such values are positively associated with prosocial attitudes and behaviors, such as fairness and caring [55]. Additionally, communal sharing in relational mode theory [56] has correlated positively with universalism and benevolence and negatively with power and achievement, while a market pricing relational mode demonstrates the opposite pattern of associations with basic human values [57].

Finally, we exploratively analyzed the correlations between communal and exchange orientations (toward an acquaintance and a close person) measured with the ECO scale with objective and subjective socioeconomic status (SES). In earlier research, Bianchi and Vohs [58] found that wealthier individuals were more likely to turn away from communal social interactions. We might, therefore, expect people with higher SES to be less communal in their relational orientations. In this project, however, we rather assumed that relational orientations are more psychological than economic. Therefore, we expected their associations with measures of SES to be weaker than the corresponding associations with psychological traits. As a result, we made no strict predictions concerning these correlations and used Bayesian factors to interpret these results (This is the deviation from preregistration suggested by a reviewer.). This study was preregistered at https://aspredicted.org/j9vk-788d.pdf.

## Materials and methods

As preregistered, we recruited 1,005 participants from Prolific Academic to complete the study in exchange for £1.30 (*n* = 499 from the US; *n* = 506 from Poland). Data collection lasted from March 7 to March 8, 2022. We excluded 83 participants from the US and 68 participants from Poland because they did not provide either valid responses to one or more attention checks (like in Study 1) or a description of their assigned person. The final sample consisted of 854 participants (see Table 1 for demographic details).

After the participants gave their informed consent, they were asked to answer questions about objective and subjective SES. The former was measured as either a monthly income (in the Polish sample) or annual income (in the American sample), using a 12-point scale, with the sixth point containing the median income for the country. The latter was measured, using MacArthur’s ladder [59]. Participants from both countries were then asked to complete six questionnaires: a 20-item version of the ECO Scale with regard to a person with whom they had a close relationship and to an unfamiliar adult person, as in Studies 1 and 2; an 18-item scale that measured self-interest and other-interest [47]; a nine-item Material Values Scale (MVS) that measured materialism [48]; the 36-item Experiences in Close Relation-Revised (ECR-R) scale [49], which measures attachment anxiety and avoidance; and (6) the Portrait Value Questionnaire (PVQ-21) [54], which measures 10 basic human values (conformity, tradition, security, benevolence, universalism, self-determination, stimulation, hedonism, achievement, and power), organized into four second-order dimensions (conservation, openness to change, self-enhancement, and self-transcendence). The ECO and MVS scales were presented with a response scale that ranged from 1 = “strongly disagree” to 5 = “strongly agree”; self- and other-interest and the ECR-R questionnaires used a 7-point Likert scale, ranging from 1 = “strongly disagree” to 7 = “strongly agree”; and the PVQ-21 used a 6-point scale from 1 = “not like me at all” to 6 = “very much like me.” The order of the scales and the order of the items within each scale were randomized (detailed information about the scales used in this study is provided in Supplementary Information).

## Results

### Confirmatory factor analysis

As a first step, we retested the proposed model of the ECO Scale, which we had already described in Studies 1 and 2, using the same methods as before. The model with four latent variables representing the exchange and communal orientation dimensions separately for the relationship with a close person and the relationship with an acquaintance was an acceptable fit to the data: RMSEA = .075, 90% CI [.073,.077], SRMR = .066, GFI = .96, CFI = 0.82, TLI = 0.81. All standardized factor loadings were significant, positive, and higher than .59. We observed significant and negative correlations between exchange orientation and communal orientation for both a close relationship, *r* = −.43, and a relationship with an acquaintance, *r* = −.43, *P*s < .001. We also observed significant correlations between the exchange orientations in two types of relationships, *r* = .35, and between the communal orientations in two types of relationships, *r* = .16, *P*s < .001. Again, we compared this model with another model that assumed that exchange orientation and communal orientation (in the relationship with an acquaintance and with a close person) represented two sides of the same construct. The fit for this model was notably poorer than for the model with separate EO and CO dimensions, RMSEA = .116, 90% CI [.114,.118], SRMR = .124, GFI = .97, CFI = 0.56, TLI = 0.54. These results again supported our hypothesized scale structure, in which exchange and communal orientations are two distinct but correlated constructs.

### Measurement invariance

Consistent with preregistration, we conducted a measurement invariance analysis with multigroup CFA to assess the psychometric equivalence of the ECO Scale across country groups in the same manner as we did in Study 1. First, we assessed the two-factor model separately for participants from Poland and the US. Then, we examined the psychometric equivalence of the ECO Scale between the two groups and tested configural, metric, and scalar invariance.

We evaluated the two-dimensional model with four latent variables separately for participants from Poland (*n* = 438) and the US (*n* = 416). Maximum likelihood CFA for the American sample yielded an acceptable model concerning most, but not all indices, RMSEA = .073, 90% CI [.070,.077], GFI = .96, CFI = 0.85, TLI = 0.84, SRMR = .068. Model fit for the Polish sample was slightly worse but still acceptable for most indices: RMSEA = .080, 90% CI [.077,.084], GFI = .97, CFI = .77, TLI = .76, SRMR = .077, supporting the configural invariance. We, therefore, performed a test of measurement invariance between participant groups concerning metric, scalar, and residual invariance (Table 6).

**Table 6:** Measurement invariance across country groups in study 3.

Type of invariance	RMSEA	GFI	CFI	TLI	ΔRMSEA	ΔCFI	ΔTLI
Unconstrained model	.075	.96	0.817	0.806	–	–	–
Configural	.077	.96	0.812	0.801	.002	.005	.005
Metric	.077	.96	0.809	0.802	<.001	.003	−.001
Scalar	.082	.96	0.776	0.774	.005	−.033	−.028
Partial scalar	.078	.96	0.796	0.794	.002	−.013	−.008

As in Study 1, the ΔRMSEA, ΔCFI, and ΔTLI were below .01 in terms of configurational and metric invariance, suggesting that the scale structure and factor loadings of the items on the latent variables did not differ between country groups. However, we again found no support for scalar invariance when we imposed restrictions on the item intercepts, implying that at least some item intercepts differed between countries. Therefore, we further investigated whether partial scalar invariance could be achieved by taking a backward-looking approach and removing restrictions on consecutive items. We found that releasing four items of the latent variable representing communal orientation in the relationship with an acquaintance and four items of the latent variable representing exchange orientation in the relationship with a close person resulted in partial scalar invariance in terms of ΔRMSEA and ΔTLI, but not ΔCFI (see Table 6). In summary, we concluded that we had replicated the results found in Study 1, which showed that the measurement model was invariant across country groups with respect to communal orientation in a relationship with a close person and with respect to exchange orientation in a relationship with an acquaintance.

### Hypotheses testing

We next examined the correlations between the dimensions of the ECO scale and related criteria. According to our preregistered hypotheses, we expected that communal orientation (toward an acquaintance and a close person) measured with the ECO Scale would be (1) positively correlated with the other-interest scale, (2) negatively correlated with the self-interest scale, (3) negatively correlated with attachment anxiety and avoidance, (4) negatively correlated with materialistic values, (5) positively correlated with conservation and self-transcendence (social focus values), and (6) negatively correlated with openness to change and self-enhancement (personal-focus values). We expected the opposite pattern of correlations for exchange orientation (toward an acquaintance and a close person), as measured with the ECO Scale. We also predicted that the same pattern of correlations would hold for both the Polish and American samples.

Our predictions have been partially confirmed (see Table 7). Communal orientation (both towards a close person and towards an acquaintance) was indeed positively correlated with other-interest. However, it did not correlate with self-interest and materialism. Exchange orientation (towards a close person and an acquaintance), on the other hand, correlated significantly and positively with self-interest and materialism, but not with other-interest. The pattern of correlations between our two dimensions and basic human values was consistent for the relationship with a close person and the relationship with an acquaintance but deviated from our preregistered hypotheses. We found that both aspects of exchange orientation correlated positively with self-enhancement but, contrary to our predictions, also correlated positively with conservation (a social orientation). In turn, the two communal orientations correlated positively with self-transcendence, but they also correlated positively with openness to change (a personal orientation value). No other correlations with basic human values were significant. Finally, the correlations with insecure attachment were only consistent for the relationship with a close person, with the exchange orientation towards a close person correlating positively and communal orientation towards a close person correlating negatively with attachment anxiety and avoidance.

**Table 7:** Descriptive statistics and correlations between variables measured in study 3.

Variable	*M*	*SD*	α	Total sample				US sample				Polish sample				Comparison of correlations in the Polish and U.S. samples (*Z*-test)			
				EO-C	EO-A	CO-C	CO-A	EO-C	EO-A	CO-C	CO-A	EO-C	EO-A	CO-C	CO-A	EO-C	EO-A	CO-C	CO-A
1. EO-C	25.43	8.27	.90	–				–				–							
2. EO-A	33.88	7.48	.89	.35^***^	–			.41^***^	–			.31^***^	–						
3. CO-C	45.11	5.58	.93	−.38^***^	−.05	–		−.31^***^	−.03	–		−.47^***^	−.06	–					
4. CO-A	29.49	7.36	.90	−.02	−.40^***^	.16^***^	–	−.03	−.31^***^	.18^***^	–	−.03	−.51^***^	.13^**^	–				
5. Self-interest	4.57	1.04	.84	.24^***^	.30^***^	.05	.02	.35^***^	.33^***^	.07	.04	.14^**^	.22^***^	.05	.03	3.16^**^	1.72	0.29	0.12
6. Other-interest	4.69	0.99	.88	−.05	.01	.31^***^	.27^***^	.07	.07	.23^***^	.27^***^	−.21^***^	−.07	.40^***^	.27^***^	4.27^***^	2.06^*^	2.70^**^	0.06
7. Materialism	3.05	0.73	.81	.20^***^	.24^***^	−.04	−.01	.24^***^	.25^***^	−.02	−.01	.19^***^	.19^***^	−.05	.01	0.81	1.04	0.55	0.07
5. Conservation	3.52	0.80	.59	.20^***^	.15^***^	.05	.05	.37^***^	.27^***^	.03	.03	.03	.03	.08	.07	5.20^***^	3.55^***^	0.86	0.61
6. Self-enhancement	3.74	0.92	.55	.21^***^	.19^***^	.05	.05	.23^***^	.27^***^	.09	.06	.18^***^	.21^***^	.00	−.01	0.75	0.90	1.30	0.99
7. Self-transcendence	4.15	0.80	.58	−.07	.05	.35^***^	.25^***^	.14^**^	.10^*^	.26^***^	.29^***^	−.28^***^	−.08	.48^***^	.27^***^	6.20^***^	2.70^**^	3.75^***^	0.28
8. Openness to change	4.07	0.84	.71	.07	.00	.15^***^	.18^***^	.03	.11^*^	.20^***^	.15^***^	.07	.05	.12^*^	.17^***^	0.57	0.83	1.22	0.33
9. Anxious attachment	3.38	1.34	.95	.24^***^	.07^*^	−.19^***^	.08^*^	.19^***^	.14^***^	−.12^*^	.05	.31^***^	−.04	−.27^***^	.14^**^	1.87	2.74^**^	2.26^*^	1.40
10. Avoidant attachment	3.39	1.16	.93	.14^***^	−.06	−.36^***^	−.11^**^	.04	−.04	−.29^***^	−.16^***^	.26^***^	−.05	−.48^***^	−.06	3.31^***^	0.22	3.23^**^	1.44
11. Income	3.91	2.83	–	−.06	.02	.03	.01	−.04	−.01	.03	.02	−.07	.01	.04	.03	0.53	0.38	0.17	0.07
BF_01_				3.92	19.49	14.29	21.73	11.93	15.79	12.93	14.48	4.76	15.99	10.68	13.98				
12. SES	5.08	1.70	–	−.04	−.04	.13^**^	.10^**^	−0.01	−.08	.13^**^	.09	−.06	−.02	.14^**^	.14^**^	0.70	0.86	0.15	0.75
BF_01_				13.29	10.42	0.01	0.24	15.88	4.27	0.40	3.03	7.77	15.09	0.18	0.22				

*Note*. *** *P* < .001, ** *P* < .01, * *P* < .05.

EO-C: exchange orientation toward a close person; EO-A: exchange orientation toward an acquaintance; CO-C: communal orientation toward a close person; CO-A: communal orientation toward an acquaintance.

In a further step, we compared the pattern of correlations found in the American and Polish samples with Fischer’s *Z*-transformation (see Table 7). The correlation between exchange orientation toward a close person and self-interest was stronger in the American sample than in the Polish one. In the Polish sample, we additionally observed a negative correlation of this ECO dimension with other-interest. In both samples, exchange orientations were positively correlated with materialism and self-enhancement, while such correlations were nonsignificant for communal orientations. Communal orientations, in turn, were correlated with self-transcendence and openness to change in both samples, although the correlations between communal orientation to a close person and self-transcendence were significantly stronger for Poles than for Americans. We observed a slightly different pattern of correlations for exchange orientation, conservation, and self-transcendence. Exchange orientation correlated significantly with conservation only in the American sample, whereas there was no such correlation in the Polish sample. Unlike our predictions, self-transcendence correlated positively with exchange orientation in the American sample, whereas the corresponding correlations were negative in the Polish group.

The pattern of correlations between insecure attachment and the dimensions of the ECO scale was also slightly different across cultures. Although exchange orientation toward a close person was positively correlated with attachment anxiety in both national samples, this correlation was stronger for Poles than for Americans. This was also true for the negative correlations between communal orientation toward a close person and both dimensions of insecure attachment. The correlations with attachment orientations for relationships with acquaintances were much weaker in both samples (Table 7).

We also analyzed the correlations between the ECO dimensions, income, and subjective SES. We found no significant correlations with personal income. Subjective SES was not correlated with exchange orientation in either sample, but it was weakly positively correlated with communal orientation (toward an acquaintance and a close person). The pattern of correlations with income and SES was the same in both national samples (Table 7).

To provide further evidence for the presence of null correlations with income and SES, we calculated Bayes factors (BF_01_) that provide a way to quantify evidence for or against null hypotheses, assuming that variables are not correlated relative to an alternative hypothesis, assuming that variables are correlated (Table 7). In most cases, BF_01_ > 10, providing strong evidence for the null correlations. For the correlations between subjective SES and communal orientation (toward a close person and an acquaintance), BF_01_ < 0.33, providing moderate-to-strong evidence for the existence of correlations. In summary, although the general pattern of results differed slightly from what we had hypothesized in the preregistration, we concluded that our findings supported the notion that exchange and communal orientations toward a particular person are two distinct—albeit correlated—constructs that indicate a more general exchange and communal orientation that people hold in relationships with others, and that these constructs showed similar validity in the Polish and American samples.

### Study 4

Having established the validity and nomological network for the dimensions of exchange and communal orientation constituting the ECO Scale, we aimed to examine its stability over time. We tested a group of Polish and American respondents twice over three weeks (American sample, T1: January 31, 2022; T2: February 22, 2023) and five weeks (Polish sample, T1: February 2, 2022; T2: March 10, 2022; This was a deviation from the preregistration. The T2 study in Poland was scheduled for the very day the Russian invasion of Ukraine began, so we decided to postpone it). We expected good test-retest reliability for the dimensions of our ECO Scale (i.e., we expected that the correlations between exchange and communal orientations toward a close person and an acquaintance measured at two time points would be high and significant). We also expected these correlations to be stronger for individuals who described the same person as either an acquaintance or a close person at both measurement times. This study was preregistered at https://aspredicted.org/jcxz-nc3z.pdf.

## Materials and methods

In line with our preregistration, we aimed to recruit 300 participants (per country) at T1 and to collect data from as many of them as possible within 48 hours at time T2. At T1, we thus recruited 601 participants from Prolific Academic in exchange for £0.75 (*n* = 279 from the US, and *n* = 322 from Poland). We excluded 48 participants who did not provide either valid responses to one or more attention checks (same as in Study 1) or a description of their assigned person. We then invited the remaining participants to the second wave of the study (*N* = 553, including the US [*n* = 254] and Poland [*n* = 299]), and a total of 549 participants filled in the questionnaire within 48 hours of the invitation. Again, we excluded those who failed to provide valid responses to one or more attention checks (same as in Study 1), leaving a final total sample of 491 participants (see Table 1 for details). Such a sample had a power of .95 to detect a correlation of .19 with a significance of .01 [60]. In both waves of the study, after providing informed consent, participants were asked to complete two questionnaires: a 20-item version of the ECO Scale with regard to a person with whom they had a close relationship and to an adult person they have recently met and about whom they could not yet say whether the relationship would develop in any way (response scale ranged from 1 = “strongly disagree” to 5 = “strongly agree”).

## Results

The correlations between the two measurement points for the two ECO dimensions in relation to a close person and an acquaintance were significant and stronger than .50 in both samples, indicating a high reliability of the measurement. Furthermore, consistent with our predictions, we found that these correlations were significantly stronger when a participant reported and rated the same person at both time points than when their rating referred to different people (Table 8).

**Table 8:** Test-retest correlations in study 4.

ECO	Total sample *N* = 491	US sample *n *= 223	PL sample *n *= 268	Same close person *n* = 253	Different close person *n* = 238	Same acquaintance *n* = 134	Different acquaintance *n* = 357	Fisher’s *Z*
EO-C	.63^***^	.69^***^	.59^***^	.74^***^	.53^***^			3.97^***^
EO-A	.56^***^	.57^***^	.55^***^			.69^***^	.52^***^	2.66^**^
CO-C	.51^***^	.49^***^	.53^***^	.72^***^	.36^***^			5.84^***^
CO-A	.52^***^	.52^***^	.53^***^			.75^***^	.43^***^	5.02^***^

*Note.* *** *P *< .001, ** *P *< .01, * *P *< .05.

EO-C: exchange orientation toward a close person; EO-A: exchange orientation toward an acquaintance; CO-C: communal orientation toward a close person; CO-A: communal orientation toward an acquaintance

## Discussion

Study 4 confirmed the test-retest reliability of the ECO scale and provided further information on its validity. The fact that the correlations for the ECO dimensions were significantly stronger when a participant reported and rated the same person at both time points than when their rating referred to different people confirms our assumption that relational orientations should be considered target-specific. In addition, the correlations for different people in the same category (close ones and acquaintances) were stronger than the correlations of the corresponding dimensions for people from different categories that we found in previous studies. This suggests that, although relational orientations are target-specific, people may have some prototypes of such orientations for different categories of people with whom they interact.

### Study 5

In the previous studies, we found that although people seemed to have relatively similar orientations toward different close people and different acquaintances, these orientations were not coherent for targets with different degrees of closeness. Based on this idea, in Study 5 we tested the hypothesis that the ECO Scale has good diagnostic criterion validity. After our participants completed the ECO Scale in relation to a close person and mere acquaintance, we presented them with a picture of an ambiguous social situation. We then asked them to imagine that this picture shows either people who knew each other well or people who hardly know each other, and to describe such a situation. After that, we asked them to rate the description they gave according to whether it reflected communal and exchange norms. We expected that high levels of communal orientation measured with the ECO Scale would predict the perception of the ambiguous social situation as more communal, while a high level of exchange orientation measured with the ECO scale would predict the perception of the same ambiguous social situation as more exchange-like. However, we hypothesized that these relationships would depend on whether the ambiguous situation involved people who knew each other well or did not know each other well. More precisely, we expected that the orientations toward a close person would be stronger predictors of perceptions of the situation involving people who knew each other, whereas the orientations toward a stranger would be stronger predictors of perceptions of the situation involving people who did not know each other. Finally, we expected to find the same pattern of results in both national samples. The study hypotheses, design, sample size, and analyzes were preregistered at https://aspredicted.org/hjhx-m4ph.pdf.

## Materials and methods

We aimed to recruit 300 participants per language version in this study (from Poland and the US). We recruited our sample from a larger pool of participants in Study 3 who passed the attention checks (*N* = 854). Five weeks after the study, participants were invited to an apparently unrelated task in exchange for £0.38. Of these, 596 completed the procedure within 3 consecutive working days (*n* = 300 from Poland; *n* = 296 from the US; 45.8% women, 53.5% men, 0.7% other; median age: 33 years, *M* = 36.17, *SD* = 13.28). Data collection lasted from April 11, 2022, to April 13, 2022. Such a sample had a power of .95 to detect an increase in R^2^ of 4% after adding interaction terms with a significance of .01. No data were excluded at this stage, and data collection was not continued after analysis.

In the first part of the study, after providing informed consent, participants were asked to complete the ECO Scale (among other questionnaires) with regard to a person with whom they had a close relationship and to an acquaintance, as in the previous studies (the response scale ranged from 1 = “strongly disagree” to 5 = “strongly agree”). The second part of the study took place 5 weeks later. After participants gave informed consent, they were presented with a picture containing two black cartoon silhouettes on a white background facing each other. In this picture, the character on the left held a paper bag with groceries, while the character on the right held nothing [61,62]. Participants were asked to look carefully and to imagine a real-life situation of a similar kind involving, depending on the condition, two people who knew each other well (*n* = 296) or who hardly knew each other (*n* = 302), and then to describe this situation in as much detail as possible. This picture was used in earlier studies [61] and was ambiguous enough to trigger communal or exchange/market interpretations, depending on the context. On the next page, participants were asked to answer four questions that measured “exchange perception” and “communal perception,” based on questions used earlier by Bohns et al. [63]. The two items used to measure exchange perception were: “To what extent does this situation feel like a business transaction?” and “To what extent do you think one person is concerned about getting something from the other in this situation?” (*r* = .45, *P* < .001). The two items measuring communal perception were: “To what extent does this situation feel like interacting with a close friend or family member?” and “To what extent is one person concerned about the other person’s needs in this situation?” (*r* = .51, *P *< .001). Participants answered the four questions in a random order, using a 7-point scale, ranging from 1 = “not at all” to 7 = “very much.”

## Results

We conducted two linear regressions, with the perception of the situation as exchange- or communal-like as dependent variables (DVs), two orientations measured with the ECO Scale concerning the close person and the stranger as independent variables, and the experimental condition as a moderator (Table 9). All variables were *Z*-scored before analysis. Concerning the communal perception, the regression model was significant, *F*(9, 586) = 25.60, *P *< .001, and accounted for R^2^ = 27.1% of the DV variance. Communal orientations toward an acquaintance and a close person were significant predictors of the communal perception of the depicted situation together with experimental manipulation, such that (1) the higher score on communal orientation led to the perception of the situation as more communal, and (2) people who were instructed to give an example of a situation involving two people who knew each other well rated it as more communal than those instructed to give an example a situation involving people who hardly knew each other. We also found a significant interaction between experimental manipulation and communal orientation toward a stranger, and a marginally significant interaction between experimental manipulation and communal orientation toward a close person. In line with our preregistered analysis plan, we thus decomposed these interactions by investigating the relationship between the ECO dimensions and communal perception separately in two experimental conditions.

**Table 9:** Predictors of perceptions of the situation as communal and exchange-related in study 5.

Predictor	Total sample				Decomposition of the interactions							
					Condition: Familiar people				Condition: Unfamiliar people			
	β	*SE*	*t*	*P*	β	*SE*	*t*	*p*	β	*SE*	*t*	*P*
DV (communal perception)												
EO-C	−.07	.04	−1.67	^†^	−.13	.05	−1.98	^*^	−.04	.07	−0.63	
EO-A	.07	.04	1.58		.07	.05	1.01		.08	.07	1.20	
CO-C	.12	.04	3.00	^**^	25	.05	4.02	^***^	.05	.06	0.80	
CO-A	.18	.04	4.45	^***^	.07	.05	1.09		.30	.07	4.61	^***^
Condition	−.45	.04	−12.99	^***^								
EO-C * Condition	.03	.04	0.68									
EO-A * Condition	.02	.04	0.49									
CO-C * Condition	−.07	.04	−1.77	^†^								
CO-A * Condition	.13	.04	3.19	^**^								
DV (exchange perception)												
EO-C	.08	.05	1.85	^†^	.20	.06	3.02	^**^	.01	.07	0.01	
EO-A	.15	.05	3.29	^***^	.05	.05	0.71		.23	.08	3.40	^***^
CO-C	−.07	.04	−1.53		−.14	.05	−2.25	^*^	−.01	.06	−0.19	
CO-A	.06	.04	1.48		.15	05	2.39	^*^	.01	.07	0.09	
Condition	.29	.04	7.58	^***^								
EO-C * Condition	−.08	.05	−1.86	^†^								
EO-A * Condition	.11	.05	2.48	^*^								
CO-C * Condition	.05	.04	1.25									
CO-A * Condition	−.06	.04	−1.34									

*Note*. *** *P *< .001, ** *P *< .01, * *P *< .05, † *P *< .1.

EO-C: exchange orientation toward a close person; EO-A: exchange orientation toward an acquaintance; CO-C: communal orientation toward a close person; CO-A: communal orientation toward an acquaintance.

When the participant’s task was to describe a situation involving people who knew each other well, the communal perception was associated with a high level of communal orientation toward a close person, and, to a lesser extent, with a low level of exchange orientation toward such a person, and the effects for orientations toward an acquaintance were nonsignificant, *F*(4, 291) = 8.52, *P *< .001, R^2^ = 10.5%. In turn, when a participant’s task was to describe a situation involving people who hardly knew each other, the only significant predictor of communal perception was the level of communal orientation toward an acquaintance, *F*(4, 297) = 6.71, *P *< .001, R^2^ = 8.3% (Table 9).

The regression model with regard to exchange perception was significant, *F*(9, 592) = 9.61, *P *< .001, and accounted for R^2^ = 20.8% of the DV variance. Exchange orientation toward an acquaintance was a significant predictor of the exchange perception of the depicted situation, together with the experimental manipulation, such that (1) the higher score for exchange orientation led to the perception of the situation as more exchange-like, and (2) people who were instructed to give an example of a situation involving two people who hardly knew each other rated it as more of an exchange than those instructed to give a situation involving people who knew each other well.

We also found a significant interaction between experimental manipulation and exchange orientation toward an acquaintance, and a marginally significant interaction between experimental manipulation and exchange orientation toward a close person. In line with the preregistered analysis plan, we decomposed this interaction by investigating the relationship between ECO dimensions and exchange perception separately for two experimental conditions. When a participant’s task was to describe a situation involving people who knew each other well, the exchange perception was associated with a high level of exchange orientation toward a close person, and, to a lesser extent, with a low level of communal orientation toward such a person, *F*(4, 291) = 8.52, *P *< .001, R^2^ = 10.5%. We also noted the unexpected positive effect of communal orientation toward a stranger on exchange perception. In turn, when the participant’s task was to describe a situation involving people who hardly know each other, the only significant predictor of exchange perception was the level of exchange orientation toward an acquaintance, *F*(4, 297) = 4.29, *P *= .002, R^2^ = 5.5% (Table 9).

## Discussion

In conclusion, Study 5 demonstrated that the dimensions of the ECO Scale had good diagnostic criterion validity. As hypothesized, high levels of communal orientation (toward both an acquaintance and a close person) predicted perceptions of an ambiguous social situation as more communal-like, while a high level of exchange orientation (toward both a stranger and a close person) predicted perceptions of the ambiguous social situation as more exchange-like. These associations were moderated by the degree of familiarity between the people depicted in the picture in a way hypothesized in the preregistration. Finally, while an unexpected positive effect of communal orientation toward a stranger on exchange perception was statistically significant, the effect size was small, and its underlying mechanisms were unclear. Consequently, this finding should be interpreted with caution, as it may reflect a statistical artifact rather than a meaningful relationship. Future research is needed to further investigate this result to determine its robustness.

### Study 6

The aim of the final Study 6 was to further test the criterion validity of the ECO scale. This time, we designed a study that captured participants’ actual behaviors as the DV rather than just measuring declarations or perceptions. We operationalized our DV as the willingness to provide assistance to a stranger (i.e., a researcher conducting studies on Prolific) in a purely communal situation, a purely exchange situation, and a situation with conflicting communal and exchange cues. More specifically, we asked some of our of participants if they would be willing to help us by participating in future studies without compensation (communal frame). Another group of participants was asked if they would be willing to participate in future studies in which they would have to compete fiercely to earn a large amount of money for themselves, but possibly at the expense of other participants (exchange frame). The remaining participants were asked if they would be willing to help us by participating in future studies in exchange for compensation that was below the usual rates paid on Prolific Academic, i.e., do not commensurate with to their time or effort (communal framework mixed with a cue that contradicts exchange orientation). Two weeks later, we sent invitations to those who had agreed to participate in these studies and checked whether they actually took part.

Overall, we predicted that high levels of communal and exchange orientations toward an acquaintance (but not toward a close person), as measured with the ECO scale, would predict both the willingness to participate in future studies and the actual participation. Furthermore, we hypothesized that these relationships would be moderated by the framing of the future study. We expected that (1) when participants were asked to help the experimenter and no compensation was offered, their behavior would be predicted only by high communal orientation toward an acquaintance; (2) when the study offered compensation that was below standard Prolific standards and was framed as helping the experimenter, a participant’s behavior would be predicted by low levels of exchange orientation toward an acquaintance, and, to a lesser extent, high levels of communal orientation toward an acquaintance; and (3) when the study was described as a highly competitive economic game played with other people, the participant’s behavior would be predicted by high levels of exchange orientation toward an acquaintance. The study hypotheses, design, sample size, and analyzes were preregistered at https://aspredicted.org/5trk-q38y.pdf.

## Materials and methods

In Study 5, we found that after adding four interaction terms (four ECO Scale dimensions X experimental manipulation) to a model with a total of nine predictors (four ECO dimensions, manipulation, and their interactions), the explained variance increased by ΔR^2^ = 1.9% for communal evaluation, and by ΔR^2^ = 2.5% for exchange-like ratings. A priori power analysis with G*Power [60] indicated that with an alpha of .05 and a conventionally assumed power of .80, a sample of 622 participants would be required to detect the weaker effect of these two. Because we planned three experimental conditions instead of two, and because we planned to use logistic regression, which requires more statistical power than linear regression with a continuous DV, we planned to double the sample size and recruit 1,244 participants.

We recruited 1,248 participants from the United Kingdom via Prolific Academic to participate in this study in exchange for £0.75. Data collection for the first part took place on April 8, 2022, and for the second part on April 22, 2022. We excluded 241 participants who either did not provide valid responses to one or more attention checks or failed to provide a description of their assigned person, leaving a final sample of 1,007 participants (see Table 2 for details). No data were excluded at this stage, and data collection was not continued after analysis.

After giving informed consent, participants were asked to complete our ECO Scale concerning a person with whom they had a close relationship and an acquaintance, as in previous studies, with a response scale that ranged from 1 = “strongly disagree” to 5 = “strongly agree”). Participants were then assigned to one of the three conditions in a between-subjects design. Participants from the first two groups were informed that, in some cases, we—as researchers—did not have sufficient financial resources to conduct research and were asked if they could help us and participate in future studies without compensation (*n* = 335), or with compensation below the usual rates paid on Prolific Academic (*n* = 334). Participants from the third group (*n* = 338) were asked if they would be willing to participate in studies in which they would have to compete strongly against one another, earning a large amount of money for themselves but possibly at the expense of other participants. All participants were then asked if they would agree to be contacted via Prolific messages to receive the invitation with a link to the study, and most agreed (*n* = 691, 68.62%). Their responses, coded as 1 = “yes” and 0 = “no,” were used as the first DV in this study. Two weeks later, following Prolific’s ethical guidelines, we selected only those participants who had agreed to take part in the study with deception (*n* = 624). We sent them a message with an invitation to the study with no compensation (*n* = 143), with low compensation (*n* = 203), or a competitive study (*n* = 278), depending on the condition to which they were assigned in the previous part of the study. Those who clicked on the link and provided their Prolific ID (*n* = 326) were then debriefed and received a small bonus of £0.20 (the same in all three conditions). This was our second DV in this study (coded as 1 = clicked, 0 = not clicked within the next 72 hours).

## Results

First, we ran our preregistered logistic regression with the declared willingness to participate in future studies as the DV, exchange and communal orientations toward a close person and an acquaintance (as measured with the ECO Scale) as independent variables, and the experimental condition as the moderator (indicator-coded, with no compensation condition as the reference point). The regression was significant, χ^2^ (14, *N* = 1,007) = 241.65, *P *< .001, Cox and Snell R^2^ = 21.3%, Nagelkerke R^2^ = 30.00% (Table 7). Consistent with our predictions, we found that communal orientation toward an acquaintance had a significant and positive effect on the willingness to help, and we found no such effect for exchange orientation toward an acquaintance. We also found a significant and positive effect of communal orientation toward a close person. The effect of exchange orientation toward a close person was nonsignificant, consistent with our predictions. Most importantly, we found that orientation toward an acquaintance (but not toward a close person) interacted with our experimental manipulation (see Table 10). Consistent with the preregistration, we therefore disaggregated these interactions by examining the links between our relational orientations and the DV separately for each experimental condition (see Table 11).

**Table 10:** Predictors of willingness to participate and actual participation in future studies in study 6.

Predictor	DV1: Willingness to participate in studies					DV2: Actual participation				
	*B*	*SE*	Wald (1)	P	Exp(B)	*B*	*SE*	Wald (1)	P	Exp(B)
EO-C	0.07	0.02	7.60	**	1.07	0.06	0.03	3.34	†	1.06
EO-A	−0.01	0.02	0.41		0.99	0.01	0.02	0.16		1.01
CO-C	0.06	0.02	10.34	**	1.06	0.06	0.02	7.38	**	1.06
CO-A	−0.03	0.02	1.94		0.97	−0.01	0.02	0.27		0.99
Condition										
Low compensation vs. no compensation	3.64	1.90	3.67	†	38.26	3.56	2.48	2.07		35.43
Competitive study vs. no compensation	3.25	2.90	1.26		25.90	4.75	2.37	4.02	*	115.58
EO-C * Condition										
EO-C * Low compensation vs. no compensation	0.03	0.03	1.13		1.03	0.02	0.03	0.65		1.02
EO-C * Competitive study vs. no compensation	0.03	0.03	0.99		1.03	−0.01	0.03	0.06		0.99
EO-A * Condition										
EO-A * Low compensation vs. no compensation	−0.05	0.03	2.68		0.95	−0.07	0.03	4.02	*	0.94
EO-A * Competitive study vs. no compensation	0.12	0.04	7.78	**	1.13	0.07	0.03	4.41	*	1.07
CO-C * Condition										
CO-C * Low compensation vs. no compensation	0.01	0.03	0.02		1.00	0.04	0.04	0.74		1.04
CO-C * Competitive study vs. no compensation	−0.07	0.05	2.23		0.93	−0.06	0.04	2.49		0.94
CO-A * Condition										
CO-A * Low compensation vs. no compensation	−0.05	0.03	3.27	†	0.95	−0.09	0.03	10.08	**	0.91
CO-A * Competitive study vs. no compensation	−0.08	0.04	4.17	*	0.93	−0.09	0.03	9.23	**	0.91

*Note*. *** *P *< .001, ** *P *< .01, * *P *< .05, † *P *< .1.

EO-C: exchange orientation toward a close person; EO-A: exchange orientation toward an acquaintance; CO-C: communal orientation toward a close person; CO-A: communal orientation toward an acquaintance.

**Table 11:** Predictors of Willingness to Participate in Future Studies in Study 6—Decomposition of the Interactions.

Predictors	DV1: Willingness to participate in studies					DV2: Actual participation				
	*B*	*SE*	Wald (1)	*P*	Exp(B)	*B*	*SE*	Wald (1)	*P*	Exp(B)
No compensation (communal frame)										
EO-C	−0.01	0.02	0.41		0.99	0.01	0.02	0.16		1.01
EO-A	−0.03	0.02	1.94		0.97	−0.01	0.02	0.27		0.98
CO-C	0.07	0.02	7.60	**	1.07	0.06	0.03	3.37	†	1.06
CO-A	0.06	0.02	10.34	**	1.06	0.06	0.02	7.38	**	1.06
Low compensation study (mixed frames)										
EO-C	0.02	0.02	0.73		1.02	0.03	0.02	2.67		1.03
EO-A	−0.08	0.02	10.71	**	0.93	−0.08	0.02	12.92	***	0.92
CO-C	0.06	0.02	8.34	**	1.07	0.10	0.03	12.95	***	1.10
CO-A	0.01	0.02	0.12		1.01	−0.03	0.02	2.99	†	0.97
Competitive study (exchange frame)										
EO-C	0.02	0.03	0.61		1.02	0.01	0.02	0.01		1.00
EO-A	0.10	0.04	5.88	*	1.10	0.05	0.02	6.77	**	1.06
CO-C	−0.01	0.04	0.03		0.99	−0.01	0.02	0.02		1.00
CO-A	−0.02	0.03	0.41		0.98	−0.03	0.02	2.27		0.97

*Note*. *** *P *< .001, ** *P *< .01, * *P *< .05, † *P *< .1.

EO-C: exchange orientation toward a close person; EO-A: exchange orientation toward an acquaintance; CO-C: communal orientation toward a close person; CO-A: communal orientation toward an acquaintance.

When we asked participants for non-contingent help (communal cue), their willingness to participate was significantly and positively associated with their communal orientation toward an acquaintance and a close person, but not with their exchange orientations, χ^2^ (4, *N* = 335) = 30.74, *P *< .001, Cox and Snell R^2^ = 8.8%, Nagelkerke R^2^ = 11.7%. When help was associated with underpayment (communal and exchange cues, mixed), a participant’s willingness to be involved in the studies was negatively associated with exchange orientation toward an acquaintance but positively associated with a communal orientation toward a close person, χ^2^ (4, *N *= 334) = 22.85, *P *< .001, Cox and Snell R^2^ = 6.6%, Nagelkerke R^2^ = 9.3%. Finally, when the studies were described as highly competitive economic games (exchange cue), a participant’s eagerness to enter was associated only with a high exchange orientation toward an acquaintance, χ^2^ (4, *N* = 338) = 13.89, *P *< .001, Cox and Snell R^2^ = 4.0%, Nagelkerke R^2^ = 8.9% (Table 11).

Second, we ran the preregistered logistic regression with actual participation in the study (clicking on the link) as the DV, relational orientations measured with the ECO Scale as independent variables, and the experimental condition as the moderator (indicator-coded, with no compensation condition as the reference point). The regression was significant, χ^2^ (14, *N* = 1,007) = 118.96, *P *< .001, although the predictors explained a smaller part of the variance, Cox and Snell R^2^ = 11.1%, Nagelkerke R^2^ = 15.6%. Regarding the willingness to participate, and consistent with our predictions, we found a significant and positive effect of communal orientation toward an acquaintance but no effect of exchange orientation toward an acquaintance. The effect of communal orientation toward a close person was positive and marginally significant. The effect of exchange orientation toward a close person was nonsignificant, consistent with our predictions. Again, we found that exchange and communal orientations toward an acquaintance (but not toward a close person) interacted with experimental manipulation. We thus disaggregated these interactions by examining the associations between our relational orientations and the DV separately for each experimental condition.

The pattern of the results was very similar to that observed for the declared willingness to participate in future studies. When we asked participants for non-contingent help (communal cue), their participation was significantly and positively associated with their communal orientation toward an acquaintance, and, to a smaller extent, toward a close person, but not with their exchange orientations, χ^2^ (4, *N* = 335) = 14.38, *P *= .006, Cox and Snell R^2^ = 4.2%, Nagelkerke R^2^ = 6.9%. When help was underpaid (communal and exchange cues, mixed), actual participation in the studies was negatively associated with exchange orientation toward an acquaintance but positively associated with a communal orientation toward a close person, χ^2^ (4, *N *= 334) = 25.35, *P *< .001, Cox and Snell R^2^ = 7.3%, Nagelkerke R^2^ = 10.2%. Finally, when the studies were described in terms of highly competitive economic games (exchange cue), actual participation was associated only with a high exchange orientation toward an acquaintance, χ^2^ (4, *N* = 338) = 16.34, *P *= .003, Cox and Snell R^2^ = 4.7%, Nagelkerke R^2^ = 6.3% (Table 11).

## Discussion

In summary, Study 6 provided evidence of the diagnostic and predictive validity of the ECO Scale. Relational orientations measured with this scale predicted not only the participant’s declared willingness to provide assistance in a situation characterized by purely communal, purely exchange, or conflicting communal and exchange cues, but also their actual behavior in these situations.

### General discussion

The current work builds on the communal and exchange relationships theory of Clark and Mills [2] and presents a framework for understanding individual differences in relational orientations towards specific individuals. It also introduces the ECO scale—a target-specific, self-report instrument that assesses the extent to which individuals tend to follow communal or exchange norms or rules in establishing and maintaining relationships with others. In six studies (total *N* = 3,252) conducted in three countries (Poland, USA, and UK), we documented that the 20-item ECO scale in two language versions (English and Polish) exhibits robust psychometric properties and is a valid and reliable measure of the exchange and communal orientations people hold toward specific individuals.

Several arguments seem to support the high quality of the ECO scale developed in this project. First, all studies were preregistered and conducted with large samples representing diverse populations. Second, we used CFAs to gather multiple sources of support, confirming that the two dimensions representing communal and exchange orientations are correlated but relatively independent. The strength of the negative correlation between the two dimensions proved similar, regardless of whether they measured relationships with a close person or with an acquaintance. However, the communal orientation toward a close person was significantly correlated with a communal orientation toward an acquaintance, whereas this correlation was somewhat weaker for the exchange orientation in these two contexts. It is possible that building and perceiving relationships in terms of communal norms or rules is more a matter of individual preferences than context specificity, whereas building and perceiving relationships based on exchange is more dependent on the context in which the relationship takes place. Third, we demonstrated the convergent and divergent validity of the ECO scale and its dimensions, by examining their associations with general communal and exchange orientations, CM (calculative mindset), social connectedness (Study 2), self- and other-interest, materialism, insecure attachment orientations, and basic human values (Study 3). Fourth, we demonstrated the high test-retest reliability of the scale (Study 4). Finally, we conducted two experiments in which we showed that the orientations measured with the ECO scale can predict people’s perceptions of ambiguous social situations in a manner consistent with either communal or exchange rules (Study 5). These orientations can also predict declared and actual helping behavior, both when monetary compensation was offered and when it was not (Study 6). Such outcomes demonstrate that relational orientations are associated with people’s perceptions of the social environment and their actual decisions and social behaviors, and that the ECO scale effectively captures individual differences in relational orientations.

Interestingly, the pattern of correlations with Schwartz’s human values turned out to be different than expected. We had predicted that the exchange orientation would correlate positively with personal-focus values and negatively with social-focus values, and that the communal orientation would show an opposite pattern of correlations. Instead, the communal orientation was positively associated with the two anxiety-free, self-expansion, and growth values (openness to change and self-transcendence), whereas the exchange orientations were positively correlated with the two anxiety-based, self-protection values (self-enhancement and conservation). These results may suggest that people high in exchange orientation are more likely to seek control over their environment—an interpretation consistent with the findings of Gasiorowska and Zaleskiewicz [61], who showed that engaging in exchange relationships can fulfill compensatory control functions when people’s sense of control is threatened. These authors demonstrated that under a control threat, participants favored exchange relationships over communal relationships, and interpreted ambiguous social interactions as similar to exchanges. Although this effect was more pronounced in relationships with acquaintances, it also existed in relationships with close people. In another study, Gasiorowska and Zaleskiewicz [64] found that experiencing a control threat increased the tendency to engage in exchange relationships among participants with higher attachment anxiety. Additionally, participants with high attachment avoidance exhibited this tendency even without a control threat. These effects suggest that involvement in exchange relationships may fulfill important psychological needs, such as serving as a buffer against attachment insecurities. This interpretation is also supported by the positive correlation between exchange orientation and insecure attachment orientations found in our Study 3. Conversely, people high in communal orientation, who are also more likely to have a secure attachment style, may be more confident in pursuing anxiety-free values that contribute to personal growth and social bonding. Importantly, however, the results were slightly different in the US and Poland (where conservation was not significantly related to exchange orientation, as it was in the US), which also prompts further research into cultural differences in this regard.

### The specificity of the ECO scale

In contrast to previous approaches to measuring communal and exchange orientations [9,32,65–67], the ECO scale allows to examine the psychological characteristics of a specific relationship with another person. Although we used examples of a close person and an acquaintance as targets when testing the scale, the scale can be used for a wide range of specific relationship targets, such as a colleague, a supervisor, a friend, and so on. Such an approach allows the investigation of a situation in which people show strong preferences for both communal and exchange relationships, depending on with whom they interact.

Crucially, the results obtained in our studies seem to explain why the relational orientation scales developed by Clark and Mills [2] were uncorrelated. Consistent with the predictions of Williamson and Clark [32], the results pertaining to our target-specific scale indicated that individuals might be simultaneously communal in close relationships and exchange-oriented in relationships with acquaintances. Furthermore, although Clark and Mills [2,4] proposed that people typically form communal relationships with family members or friends and exchange relationships with those with whom they do not have strong ties, our results indicate that some individuals may be willing to form an exchange relation with close others, contradicting the classical understanding of the nature of the two relationships.

### Contribution of the present research to the understanding of social relationships

Using the ECO scale may improve the ability to predict behavior, cognitions, and emotions related to relationships with diverse individuals. This ability is crucial when considering that people’s relationships vary considerably due to both situational factors and relatively stable individual differences. Accurately measuring relational orientations can also help us understand the challenges people might have when interacting with others. It is adaptive to adjust one’s behavior to different relational contexts, i.e., to behave communally in close relationships, as it improves their quality and duration [20], while applying an exchange-based logic in relationships with more distant people (e.g., business partners). Our scale can identify people who fail to adapt to the relational context or who exhibit extreme levels of exchange or communal orientation in their relationships, regardless of the actual level of closeness. For example, someone might approach a person they met in a professional context with an unusually high level of communal orientation, develop unrealistic social expectations in the context of purely transactional interactions and put themselves at greater risk of exploitation (e.g., in work relationships) or relational dissatisfaction (e.g., in an interaction with a therapist). Such people may also expend too much emotional energy building relationships with people who do not want to form close connections with them. Conversely, an individual may apply an exchange logic to relationships with people close to them, which could reduce both parties’ satisfaction with the relationship, make the interactions more superficial, and reduce the stability of the relationship over time [17,18,67,68]. Therefore, the ECO scale can potentially be used to identify problems in close relationships that might due to a person’s tendency to use exchange rules when interacting with close others.

The scale also appears to be useful in examining the causes of incompatibility between partners in different types of relationships, as evidenced by adherence to different relationship principles. An individual characterized by a more communal (exchange) orientation may perceive an ambiguous situation with the same formal characteristics as more communal (exchange) than individuals who score lower on the communal (exchange) orientation. Such divergent perceptions may further influence people’s expectations of the other party’s behavior, trigger misunderstandings within a dyad, and consequently reduce their satisfaction with relationship outcomes and possibly their personal well-being.

## Limitations and generalizability constraints

A limitation of the studies described in this article is that, although we designed our scale to be relevant to each relationship target, we used only two exemplars in all four studies: a close person (defined as a friend, life partner, or adult child) and an acquaintance (defined as an adult whom the participant has recently met and cannot yet say whether or not this relationship will develop in any way). Future studies should examine a broader range of relationships, such as those with spouses or siblings, distant relatives, best friends, work colleagues, business partners, or strangers. In this context, we see the need to test the scale’s validity further, especially its predictive and incremental aspects.

Furthermore, the validation of the ECO Scale was based on data from online panels. Although the quality of data collected in online labor markets has been questioned, research has shown that data sourced from Prolific Academic are valid and equivalent to data collected with traditional methods [69]. Though we collected behavioral data in the last experiment, most of our studies were based on self-reports. Therefore, the extent to which they reflected actual attitudes, judgments, and preferences is uncertain. Important research aims for the future might thus be to collect data from participants who are not members of online panels, use behavioral measures, and investigate real-life behavior to test the scale’s construct validity.

Our data were collected during and shortly after the second and third waves of the COVID-19 outbreak, which may have affected participants’ emotional well-being and other psychological constructs studied, including relational orientations. For instance, some participants reported being unable to recall anyone they had met recently because they had not left home or had only contacted others online. Therefore, it would be valuable to investigate whether the challenges that characterize the time frame of our study, such as the experience of uncertainty, illness, and social distancing, affect relationship orientations.

Although we do not think age or ethnicity affects relational orientations, it is important to note that our samples were younger than the general population. Additionally, although the ethnic structure of the Polish samples was similar to that of the Polish population, the American sample included an overrepresentation of White participants (see Table 1). Additionally, although we found some cross-cultural differences, both in terms of the structure of our focal construct (resulting in partial scalar invariance) and in the pattern of correlates between exchange and communal orientations and basic human values, the specific reasons behind these differences remain unknown. It seems plausible that individual differences in relational orientations are exacerbated by cultural factors, aligning with findings of Triandis [70]. He observed that communal orientation prevails in close relationships within collectivistic cultures, whereas in individualistic cultures, such relationships may also be shaped by exchange orientation. However, nationals from countries in which we recruited our participants score relatively high on the dimensions of individualism and masculinity [71]. Future studies could investigate cultural differences in relational orientations measured with the ECO Scale in countries that differ more significantly in individualism and masculinity. It is possible that in countries low in masculinity (e.g., Nordic countries), where the division of emotional roles between women and men is less discrepant than in countries high in masculinity (e.g., Italy), the differences between genders concerning their relational orientations would be much smaller. It would also be interesting to test whether an exchange orientation is more prevalent in highly individualistic societies (e.g., Canada, the US, and Australia) than in more collectivistic countries (e.g., Chile, Greece).

## Supporting information

S1 File The ECO scale, english version, polish version for women (Skala ECO: wersja polska żeńska), polish version for men (Skala ECO: wersja polska męska). (DOCX)

S2 File Auxiliary information about methods. (DOCX)

S1 Table Unstandardized and standardized factor loadings and discrimination indices for the two-dimensional model (Initial Item Pool; CFA, Study 1). (DOCX)

S2 Table Unstandardized and standardized factor loadings and discrimination indices for the two-dimensional model (Final Item Pool; CFA, Study 1). (DOCX)
