# Supplementary material for: Exchange and Communal Orientations (ECO) scale: The construction and validation of a method to measure target-specific relational orientations
Source: PLoS One. 2025 Jun 3;20(6):e0325232. doi: 10.1371/journal.pone.0325232 (PMC12132953; doi:10.1371/journal.pone.0325232)
Supplement: S1 File — (DOCX) [file pone.0325232.s001.docx]

**The ECO Scale English version**

Please think of [*specification of a person*]

Please [*if the person is known:* write the name of this person below and] briefly describe who this person is.

Who is this person for you?

------next page-------

Please read and answer the following statements relating to the person you indicated on the previous page. We remind you that [*if the person is known*: this person's name is {$name} and you described them in following way: {$description}.

Answer scale:

(1) strongly disagree

(2) disagree

(3) neither agree nor disagree

(4) agree

(5) strongly agree

1. In the relationship with this person, the feeling of closeness and care are more important to me than any material benefits
2. I would help this person, even if it was costly to me
3. Buying this person a gift would make me happy, even if I knew that this person is not planning to buy one for me
4. I would help this person, even if it was difficult for me
5. I know that me and this person can always count on each other
6. I would pay attention to what this person needs at any given moment
7. I know that if I was in need, I could always count on this person
8. I like to make this person happy
9. I would gladly give my money or belongings to this person if I knew they needed it
10. I would love to see this person, even for no particular reason
11. When starting a joint activity with this person, I would like to know how I would benefit
12. Starting a joint activity with this person, I would like to know from the start what I will get out of it
13. I would pay a lot of attention to equal division of tasks
14. If we were to divide something (e.g., money), I would pay attention that I do not lose out
15. If this person broke or destroyed something of mine, I would expect them to make it up to me financially
16. If I were to do something together with this person, I would find it important that we put the same amount of effort into it
17. I would expect this person to swiftly repay me the costs I incurred
18. I would make sure that the tasks are equally divided between me and this person
19. If we worked together, I would make sure that this person works at least as hard as me
20. If I were to give something to this person, I would like to be sure that it would be reciprocated

**ECO Scale – Polish version for women**

**Skala ECO – wersja polska żeńska**

Pomyśl o [*doprecyzowanie osoby*]

[*Jeśli osoba jest znana:* Wpisz poniżej imię tej osoby i] opisz w kilku słowach, kim ta osoba jest.

Kim ta osoba jest dla Ciebie?

------next page-------

Przeczytaj poniższe stwierdzenia i zaznacz na skali, w jakim stopniu każde z nich opisuje Twoje relacje ze wskazaną przez Ciebie osobą. Przypominamy, że [*Jeśli osoba jest znana* : ta osoba ta ma na imię {$imię} i] opisałaś ją w następujący sposób: {$*opis*}

Skala odpowiedzi:

(1) zdecydowanie nie

(2) raczej nie

(3) ani tak, ani nie

(4) tak

(5) zdecydowanie tak

1. Poczucie bliskości i troski w relacji z tą osobą są dla mnie ważniejsze niż jakiekolwiek korzyści materialne
2. Pomogłabym tej osobie, nawet jeśli byłoby to dla mnie kosztowne
3. Sprawiłoby mi przyjemność kupienie tej osobie prezentu, nawet gdybym wiedziała, że ta osoba nie planuje kupienia prezentu dla mnie
4. Pomogłabym tej osobie, nawet jeśli byłoby to dla mnie trudne
5. Wiem, że ja i ta osoba zawsze możemy na siebie liczyć
6. Zwracałabym uwagę na to, czego w danym momencie potrzebuje ta osoba
7. Wiem, że gdybym znalazła się w potrzebie zawsze mogłabym liczyć na tą osobę
8. Lubię uszczęśliwiać tę osobę.
9. Chętnie oddałabym swoje pieniądze lub rzeczy tej osobie, jeśli wiedziałabym, że ona tego potrzebuje
10. Chętnie spotykałabym się z tą osobą, nawet bez konkretnego powodu
11. Rozpoczynając wspólne działanie z tą osobą, chciałabym wiedzieć, jaką wyciągnę z tego korzyść dla siebie
12. Rozpoczynając jakieś działanie razem z tą osobą, chciałabym od początku wiedzieć, co będę z tego miała
13. Przykładałabym dużo uwagi do równego podziału zadań
14. Jeśli mielibyśmy się czymś podzielić (np. pieniędzmi), to zwracałabym uwagę na to, czy ja nie jestem na tym stratna
15. Gdyby ta osoba popsuła lub zniszczyła jakąś moją rzecz, to oczekiwałabym, że mi to finansowo wynagrodzi
16. Jeśli miałabym coś robić razem z tą osobą, to byłoby dla mnie ważne, żebyśmy wkładali w to tyle samo wysiłku
17. Oczekiwałabym od tej osoby szybkiej rekompensaty za poniesione przeze mnie koszty
18. Dbałabym o to, aby zadania były równo podzielone między mnie i tę osobę
19. Gdybyśmy wspólnie pracowali, to pilnowałabym, aby ta osoba pracowała co najmniej tak samo intensywnie, jak ja
20. Dając coś tej osobie, chciałabym mieć pewność, że mój gest zostanie odwzajemniony

**ECO Scale – Polish version for men**

**Skala ECO – wersja polska męska**

Pomyśl o [*doprecyzowanie osoby*]

[*Jeśli osoba jest znana:* Wpisz poniżej imię tej osoby i] opisz w kilku słowach, kim ta osoba jest.

Kim ta osoba jest dla Ciebie?

------next page-------

Przeczytaj poniższe stwierdzenia i zaznacz na skali, w jakim stopniu każde z nich opisuje Twoje relacje ze wskazaną przez Ciebie osobą. Przypominamy, że [*Jeśli osoba jest znana* : ta osoba ta ma na imię {$imię} i] opisałeś ją w następujący sposób: {$*opis*}

Skala odpowiedzi:

(1) zdecydowanie nie

(2) raczej nie

(3) ani tak, ani nie

(4) tak

(5) zdecydowanie tak

1. Poczucie bliskości i troski w relacji z tą osobą są dla mnie ważniejsze niż jakiekolwiek korzyści materialne
2. Pomógłbym tej osobie, nawet jeśli byłoby to dla mnie kosztowne
3. Sprawiłoby mi przyjemność kupienie tej osobie prezentu, nawet gdybym wiedział, że ta osoba nie planuje kupienia prezentu dla mnie
4. Pomógłbym tej osobie, nawet jeśli byłoby to dla mnie trudne
5. Wiem, że ja i ta osoba zawsze możemy na siebie liczyć
6. Zwracałbym uwagę na to, czego w danym momencie potrzebuje ta osoba
7. Wiem, że gdybym znalazł się w potrzebie zawsze mógłbym liczyć na tą osobę
8. Lubię uszczęśliwiać tę osobę.
9. Chętnie oddałbym swoje pieniądze lub rzeczy tej osobie, jeśli wiedziałbym, że ona tego potrzebuje
10. Chętnie spotykałbym się z tą osobą, nawet bez konkretnego powodu
11. Rozpoczynając wspólne działanie z tą osobą, chciałbym wiedzieć, jaką wyciągnę z tego korzyść dla siebie
12. Rozpoczynając jakieś działanie razem z tą osobą, chciałbym od początku wiedzieć, co będę z tego miał
13. Przykładałbym dużo uwagi do równego podziału zadań
14. Jeśli mielibyśmy się czymś podzielić (np. pieniędzmi), to zwracałbym uwagę na to, czy ja nie jestem na tym stratny
15. Gdyby ta osoba popsuła lub zniszczyła jakąś moją rzecz, to oczekiwałbym, że mi to finansowo wynagrodzi
16. Jeśli miałbym coś robić razem z tą osobą, to byłoby dla mnie ważne, żebyśmy wkładali w to tyle samo wysiłku
17. Oczekiwałbym od tej osoby szybkiej rekompensaty za poniesione przeze mnie koszty
18. Dbałbym o to, aby zadania były równo podzielone między mnie i tę osobę
19. Gdybyśmy wspólnie pracowali, to pilnowałbym, aby ta osoba pracowała co najmniej tak samo intensywnie, jak ja
20. Dając coś tej osobie, chciałbym mieć pewność, że mój gest zostanie odwzajemniony
