# Supplementary material for: Exchange and Communal Orientations (ECO) scale: The construction and validation of a method to measure target-specific relational orientations
Source: PLoS One. 2025 Jun 3;20(6):e0325232. doi: 10.1371/journal.pone.0325232 (PMC12132953; doi:10.1371/journal.pone.0325232)
Supplement: S2 File — (DOCX) [file pone.0325232.s002.docx]

**S4. Auxiliary information about methods**

**Study 2**

**General communal orientation (Clark & Mills, 2012)**

Cronbach’s α = .85, *M* = 3.65, *SD* = 0.60

Please read the statements below and evaluate whether they are characteristic of uncharacteristic of you using a scale from 1 = extremely uncharacteristic of me to 5 = extremely characteristic of me

- It bothers me when other people neglect my needs.

- When making a decision, I take other people’s needs and feelings into account.

- {reverse} I’m not especially sensitive to other people’s feelings.

- {reverse} I don’t consider myself to be a particularly helpful person.

- I believe people should go out of their way to be helpful.

- {reverse} I don’t especially enjoy giving others aid.

- I expect people I know to be responsive to my needs and feelings.

- I often go out of my way to help another person.

- {reverse} I believe it’s best not to get involved taking care of other people’s personal needs.

- I’m not the sort of person who often comes to the aid of others.

- When I have a need, I turn to others I know for help.

- {reverse} When people get emotionally upset, I tend to avoid them.

- {reverse} People should keep their troubles to themselves.

- When I have a need that others ignore, I’m hurt.

**General exchange orientation (Clark & Mills, 2012)**

Cronbach’s α = .73, *M* = 2.81, *SD* = 0.63

Please read the statements below and evaluate whether they are characteristic of uncharacteristic of you using a scale from 1 = extremely uncharacteristic of me to 5 = extremely characteristic of me

- When I give something to another person, I generally expect something in return.

- When someone buys me a gift, I try to buy that person as comparable a gift as possible.

- {reverse} I don’t think people should feel obligated to repay others for favors.

- {reverse} I wouldn’t feel exploited if someone failed to repay me for a favor.

- {reverse} I don’t bother to keep track of benefits I have given others.

- When people receive benefits from others, they ought to repay those others right away.

- It’s best to make sure things are always kept ‘even’ between two people in a relationship.

- I usually give gifts only to people who have given me gifts in the past.

- When someone I know helps me out on a project, I don’t feel I have to pay them back.

**Calculative Mindset scale (Kim et al., 2021)**

Cronbach’s α = .90, *M* = 2.02, *SD* = 0.75

Please read the statements below and evaluate whether you agree with them or not

- (1) strongly disagree

- (2) disagree

- (3) neither agree nor disagree

- (4) agree

- (5) strongly agree

- I calculate the value of my relationships with others.

- I see social situations (relationships) as a potential source of monetary gain.

- I put a different dollar value on time I spent with each individual.

- I tend to convert everything into numbers (e.g., time, trust, love, money)for cost-benefit analysis.

- I see all of my social relationships in terms of who can benefit me the most.

- I only keep the friendships that I view as “valuable.”

- I keep track of every social transaction.

- Before I befriend someone, I mentally assess the benefit to myself of the friendships.

- I help someone based on the expected value of the relationship.

- I cut my social relationships which I have analyzed will be not beneficial to future.

**UBC Social Connection scale (Lok & Dunn, 2022)**

Cronbach’s α = .95, *M* = 4.51, *SD* = 1.35

Please think about how you feel usually. To what extent do each of the following statements describe how you feel?

- (1) strongly disagree

- (2) disagree

- (3) slightly disagree

- (4) neither agree nor disagree

- (5) slightly agree

- (6) agree

- (7) strongly agree

- {reverse} I feel distant from people.

- {reverse} I don’t feel related to most people.

- {reverse} I feel like an outsider.

- I feel like I was able to connect with other people.

- {reverse} I feel disconnected from the world around me.

- I feel close to people.

- I see people as friendly and approachable.

- I am in tune with the world.

- I feel accepted by others.

- I have a sense of belonging.

- I feel a strong bond with other people.

**Study 3**

**Income**

What is your average personal income per month after tax) in USD?

- 0-1.000

- 1.001-2.000

- 2.001-3.000

- 3.001-4.000

- 4.001-5.000

- 5.001-6.000

- 6.001-7.000

- 7.001-8.000

- 8.001-9.000

- 9.001-10.000

- 10.000-15.000

- above 15.000

**Socio-economic status (Adler et al., 2000)**

Think of this ladder as representing where people stand in the society. At the top (10) of the ladder are the people who are the best off - those who have the most money, the most education and the most respected jobs. At the bottom (1) are the people who are the worst off - who have the least money, least education, and the least respected jobs or no job. The higher up you are on this ladder, the closer you are to the people at the very top; the lower you are, the closer you are to the people at the very bottom.

**Where would you place yourself on this ladder compared to other people in the US?  (1-10)**

**ECR-R (Fraley et al., 2000)**

The following statements concern how you feel in emotionally intimate relationships e.g., close friends, family, romantic partners). We are interested in how you generally experience relationships, not just in what is happening in a current relationship. Respond to each statement to indicate how much you agree or disagree with the statement.

Again, the following items refer to close others in general.

- (1) strongly disagree

- (2) disagree

- (3) slightly disagree

- (4) neither agree nor disagree

- (5) slightly agree

- (6) agree

- (7) strongly agree

- I'm afraid that I will lose other's love.

- I often worry that others will not want to stay with me.

- I often worry that others doesn't really love me.

- I worry that close others won't care about me as much as I care about them.

- I often wish that others feelings for me were as strong as my feelings for them.

- I worry a lot about my relationships.

- When my close person is out of sight, I worry that he or she might become interested in someone else.

- When I show my feelings for others, I'm afraid they will not feel the same about me.

- I rarely worry about others leaving me.

- My close ones make me doubt myself.

- I do not often worry about being abandoned.

- I find that other(s) don't want to get as close as I would like.

- Sometimes my close ones change their feelings about me for no apparent reason.

- My desire to be very close sometimes scares people away.

- I'm afraid that once a somebody gets to know me, he or she won't like who I really am.

- It makes me mad that I don't get the affection and support I need from others.

- I worry that I won't measure up to other people.

- My close ones only seem to notice me when I'm angry.

- I prefer not to show others how I feel deep down.

- I feel comfortable sharing my private thoughts and feelings with my close ones.

- I find it difficult to allow myself to depend on others.

- I am very comfortable being close to others.

- I don't feel comfortable opening up to others

- I prefer not to be too close to others.

- I get uncomfortable when another person wants to be very close.

- I find it relatively easy to get close to others.

- It's not difficult for me to get close to others.

- I usually discuss my problems and concerns with others.

- It helps to turn to my close ones in times of need.

- I tell my close ones just about everything.

- I talk things over with my close ones.

- I am nervous when others get too close to me.

- I feel comfortable depending on my close ones.

- I find it easy to depend on close ones.

- It's easy for me to be affectionate with my close ones.

- My close ones really understand me and my needs.

**The Portrait Value Questionnaire PVQ-21 (Schwartz et al., 2001)**

Here we briefly describe some people. Please read each description and think about how much each person is or is not like you. Indicate the box to the right that shows how much the person in the description is like you.

- (1) not like me at all

- (2) not like me

- (3) a little like me

- (4) somewhat like me

- (5) like me

- (6) very much like me

- Thinking up new ideas and being creative is important to her. She likes to do things in her own original way.

- It is important to her to be rich. She wants to have a lot of money and expensive things.

- She thinks it is important that every person in the world be treated equally. She believes everyone should have equal opportunities in life.

- It's very important to her to show her abilities. She wants people to admire what she does.

- It is important to her to live in secure surroundings. She avoids anything that might endanger her safety.

- She thinks it is important to do lots of different things in life. She always looks for new things to try.

- She believes that people should do what they're told. She thinks people should follow rules at all times, even when no-one is watching.

- It is important to her to listen to people who are different from her. Even when she disagrees with them, she still wants to understand them.

- She seeks every chance she can to have fun. It is important to her to do things that give her pleasure.

- It is important to her to make her own decisions about what she does. She likes to be free to plan and to choose her activities for herself.

- It's very important to her to help the people around her. She wants to care for their well-being.

- Being very successful is important to her. She likes to impress other people.

- It is very important to her that her country be safe. She thinks the state must be on watch against threats from within and without.

- She likes to take risks. She is always looking for adventures.

- It is important to her always to behave properly. She wants to avoid doing anything people would say is wrong.

- It is important to her to be in charge and tell others what to do. She wants people to do what she says.

- It is important to her to be loyal to her friends. She wants to devote herself to people close to her.

- She strongly believes that people should care for nature. Looking after the environment is important to her.

- She thinks it is best to do things in traditional ways. It is important to her to keep up the customs she has learned.

- Enjoying life’s pleasures is important to her. She likes to ‘spoil’ herself.

- It is important to her to be humble and modest. She tries not to draw attention to herself.

**Materialism (Richins & Dawson, 1992)**

Please indicate your level of agreement with the following statements:

- (1) strongly disagree

- (2) disagree

- (3) neither agree nor disagree

- (4) agree

- (5) strongly agree

- I admire people who own expensive homes, cars, and clothes.

- The things I own say a lot about how well I’m doing in life.

- I like to own things that impress people.

- I try to keep my life simple, as far as possessions are concerned.

- Buying things gives me a lot of pleasure.

- I like a lot of luxury in my life.

- My life would be better if I owned certain things I don’t have.

- I’d be happier if I could afford to buy more things.

- It sometimes bothers me quite a bit that I can’t afford to buy all the things I’d like.

**Self-interest / Other-interest scale** (Gerbasi & Prentice, 2013)

Please indicate your level of agreement with the following statements:

- (1) strongly disagree

- (2) disagree

- (3) slightly disagree

- (4) neither agree nor disagree

- (5) slightly agree

- (6) agree

- (7) strongly agree

- I look for opportunities to achieve higher social status.
- I am constantly looking for ways to get ahead.
- Hearing others praise me is something I look forward to.
- I try to make sure others know about my successes.

- Success is important to me.
- I keep an eye out for my interests.
- Having a lot of money is one of my goals in life.
- Getting good grades is near the top of my priorities.
- I am constantly looking out for what will make me happy.

- I am constantly looking for ways for my classmates to get ahead.
- Hearing others praise my colleagues is something I look forward to.
- I look for opportunities to help people I know achieve higher social status.

- I try to help my colleagues by telling other people about their successes.
- I want to help people I know to do well in their courses or work.
- I keep an eye out for other’s interests.
- The success of my friends is important to me.
- I look out for ways for my friends to have more money.
- It is important to me that others are happy.
