# Supplementary material for: Exchange and Communal Orientations (ECO) scale: The construction and validation of a method to measure target-specific relational orientations
Source: PLoS One. 2025 Jun 3;20(6):e0325232. doi: 10.1371/journal.pone.0325232 (PMC12132953; doi:10.1371/journal.pone.0325232)
Supplement: S1 Table — (DOCX) [file pone.0325232.s003.docx]

**S2 Table. Unstandardized and standardized factor loadings and discrimination indices for the second model CFA (initial item pool; Study 1)**

| Item number | b | se | Z | 95CI lower limit | 95CI upper limit | β | Item-rest correlation |
| --- | --- | --- | --- | --- | --- | --- | --- |
| Exchange orientation - close person | | | | | | |  |
| 1 | 1.00 | 0.00 |  | 1.00 | 1.00 | 0.58 | 0.55 |
| 2 | 1.31 | 0.09 | 14.92 | 1.13 | 1.48 | 0.76 | 0.73 |
| 3 | 1.18 | 0.08 | 14.78 | 1.02 | 1.33 | 0.73 | 0.71 |
| 4 | 1.35 | 0.08 | 16.08 | 1.18 | 1.51 | 0.79 | 0.77 |
| 5 | 1.23 | 0.09 | 14.08 | 1.06 | 1.40 | 0.72 | 0.69 |
| 6 | 1.14 | 0.08 | 13.53 | 0.98 | 1.31 | 0.80 | 0.77 |
| 7 | 1.29 | 0.09 | 14.57 | 1.12 | 1.47 | 0.74 | 0.72 |
| 8 | 1.30 | 0.09 | 15.06 | 1.13 | 1.46 | 0.82 | 0.79 |
| 9 | 1.50 | 0.10 | 14.61 | 1.30 | 1.70 | 0.76 | 0.74 |
| 10 | 1.25 | 0.09 | 14.33 | 1.08 | 1.42 | 0.77 | 0.75 |
| 11 | 1.28 | 0.10 | 12.77 | 1.08 | 1.48 | 0.53 | 0.51 |
| 12 | 1.25 | 0.09 | 14.04 | 1.08 | 1.43 | 0.72 | 0.70 |
| 13 | 1.00 | 0.10 | 10.54 | 0.81 | 1.19 | 0.51 | 0.51 |
| 14 | 1.21 | 0.09 | 13.53 | 1.04 | 1.39 | 0.79 | 0.77 |
| 15 | 1.14 | 0.07 | 15.84 | 1.00 | 1.28 | 0.73 | 0.71 |
| 16 | 0.69 | 0.09 | 7.92 | 0.52 | 0.86 | 0.31 | 0.31 |
| 17 | 1.39 | 0.09 | 15.66 | 1.22 | 1.57 | 0.80 | 0.77 |
| 18 | 1.28 | 0.09 | 13.59 | 1.10 | 1.46 | 0.62 | 0.61 |
| 19 | 1.16 | 0.08 | 14.57 | 1.00 | 1.32 | 0.77 | 0.75 |
| 20 | 1.15 | 0.08 | 13.94 | 0.99 | 1.31 | 0.68 | 0.65 |
| Communal orientation - close person | | | | | | |  |
| 21 | 1.00 | 0.00 |  | 1.00 | 1.00 | 0.73 | 0.69 |
| 22 | 1.02 | 0.04 | 25.79 | 0.95 | 1.10 | 0.74 | 0.70 |
| 23 | 0.86 | 0.05 | 16.56 | 0.76 | 0.97 | 0.59 | 0.58 |
| 24 | 0.67 | 0.05 | 12.98 | 0.57 | 0.77 | 0.49 | 0.49 |
| 25 | 1.05 | 0.05 | 21.34 | 0.96 | 1.15 | 0.69 | 0.65 |
| 26 | 0.92 | 0.05 | 17.40 | 0.81 | 1.02 | 0.59 | 0.56 |
| 27 | 0.71 | 0.05 | 15.51 | 0.62 | 0.80 | 0.54 | 0.51 |
| 28 | 0.86 | 0.06 | 15.42 | 0.75 | 0.97 | 0.58 | 0.57 |
| 29 | 0.76 | 0.06 | 13.27 | 0.65 | 0.87 | 0.49 | 0.46 |
| 30 | 0.94 | 0.05 | 19.35 | 0.84 | 1.03 | 0.66 | 0.62 |
| 31 | 0.98 | 0.05 | 20.15 | 0.88 | 1.07 | 0.65 | 0.61 |
| 32 | 0.66 | 0.06 | 11.97 | 0.55 | 0.77 | 0.37 | 0.35 |
| 33 | 0.84 | 0.05 | 15.54 | 0.73 | 0.94 | 0.57 | 0.56 |
| 34 | 0.40 | 0.06 | 7.13 | 0.29 | 0.52 | 0.26 | 0.24 |
| 35 | 0.93 | 0.05 | 18.06 | 0.82 | 1.03 | 0.63 | 0.62 |
| 36 | 0.64 | 0.05 | 12.92 | 0.55 | 0.74 | 0.51 | 0.48 |
| 37 | 0.59 | 0.05 | 12.09 | 0.49 | 0.68 | 0.44 | 0.40 |
| 38 | 0.84 | 0.05 | 15.70 | 0.74 | 0.95 | 0.55 | 0.53 |
| 39 | 0.91 | 0.05 | 19.99 | 0.82 | 1.00 | 0.66 | 0.64 |
| 40 | 0.96 | 0.05 | 18.57 | 0.86 | 1.06 | 0.63 | 0.60 |

Table S1 continued. Unstandardized and standardized factor loadings and discrimination indices for the second model CFA (initial item pool; Study 1)

| Item number | b | se | | Z | 95CI lower limit | 95CI upper limit | β | Item-rest correlation |
| --- | --- | --- | --- | --- | --- | --- | --- | --- |
| Exchange orientation – an acquaintance | | | | | | | |  |
| 1 | 1.00 | 0.00 |  | | 1.00 | 1.00 | 0.57 | 0.53 |
| 2 | 1.33 | 0.09 | 15.47 | | 1.16 | 1.50 | 0.64 | 0.62 |
| 3 | 1.20 | 0.07 | 16.93 | | 1.06 | 1.34 | 0.67 | 0.64 |
| 4 | 1.20 | 0.08 | 15.52 | | 1.04 | 1.35 | 0.71 | 0.68 |
| 5 | 1.45 | 0.08 | 17.32 | | 1.29 | 1.61 | 0.74 | 0.71 |
| 6 | 1.12 | 0.07 | 16.07 | | 0.98 | 1.26 | 0.70 | 0.67 |
| 7 | 1.14 | 0.08 | 14.59 | | 0.99 | 1.29 | 0.67 | 0.66 |
| 8 | 1.23 | 0.08 | 16.09 | | 1.08 | 1.38 | 0.72 | 0.69 |
| 9 | 1.22 | 0.08 | 15.23 | | 1.06 | 1.38 | 0.67 | 0.64 |
| 10 | 1.12 | 0.07 | 15.26 | | 0.98 | 1.27 | 0.67 | 0.65 |
| 11 | 0.84 | 0.07 | 12.76 | | 0.71 | 0.97 | 0.54 | 0.52 |
| 12 | 1.13 | 0.08 | 14.66 | | 0.98 | 1.28 | 0.68 | 0.67 |
| 13 | 0.99 | 0.09 | 11.60 | | 0.83 | 1.16 | 0.52 | 0.51 |
| 14 | 1.17 | 0.08 | 15.45 | | 1.02 | 1.31 | 0.70 | 0.68 |
| 15 | 1.14 | 0.08 | 14.59 | | 0.99 | 1.30 | 0.67 | 0.65 |
| 16 | 0.68 | 0.08 | 9.02 | | 0.53 | 0.82 | 0.37 | 0.37 |
| 17 | 1.22 | 0.08 | 15.48 | | 1.07 | 1.37 | 0.67 | 0.64 |
| 18 | 1.16 | 0.08 | 13.77 | | 1.00 | 1.33 | 0.63 | 0.62 |
| 19 | 1.20 | 0.08 | 15.51 | | 1.05 | 1.35 | 0.65 | 0.63 |
| 20 | 1.02 | 0.07 | 14.14 | | 0.88 | 1.16 | 0.56 | 0.53 |
| Communal orientation – an acquaintance | | | | | | | |  |
| 21 | 1.00 | 0.00 |  | | 1.00 | 1.00 | 0.69 | 0.65 |
| 22 | 1.04 | 0.04 | 24.81 | | 0.96 | 1.12 | 0.69 | 0.66 |
| 23 | 0.77 | 0.05 | 15.32 | | 0.67 | 0.87 | 0.59 | 0.56 |
| 24 | 0.65 | 0.05 | 13.86 | | 0.56 | 0.74 | 0.53 | 0.50 |
| 25 | 0.94 | 0.05 | 20.31 | | 0.85 | 1.03 | 0.69 | 0.65 |
| 26 | 0.86 | 0.05 | 17.86 | | 0.77 | 0.95 | 0.64 | 0.60 |
| 27 | 0.63 | 0.05 | 13.61 | | 0.54 | 0.72 | 0.44 | 0.42 |
| 28 | 0.74 | 0.05 | 14.75 | | 0.64 | 0.84 | 0.58 | 0.55 |
| 29 | 0.77 | 0.05 | 15.63 | | 0.67 | 0.87 | 0.56 | 0.53 |
| 30 | 0.97 | 0.05 | 18.69 | | 0.87 | 1.07 | 0.64 | 0.61 |
| 31 | 0.91 | 0.06 | 16.61 | | 0.80 | 1.02 | 0.57 | 0.54 |
| 32 | 0.68 | 0.05 | 13.49 | | 0.58 | 0.78 | 0.48 | 0.45 |
| 33 | 0.71 | 0.05 | 14.51 | | 0.62 | 0.81 | 0.57 | 0.54 |
| 34 | 0.40 | 0.05 | 7.43 | | 0.29 | 0.50 | 0.28 | 0.27 |
| 35 | 0.95 | 0.05 | 19.83 | | 0.86 | 1.05 | 0.68 | 0.64 |
| 36 | 0.69 | 0.05 | 13.19 | | 0.59 | 0.79 | 0.47 | 0.45 |
| 37 | 0.57 | 0.06 | 10.44 | | 0.46 | 0.68 | 0.39 | 0.37 |
| 38 | 0.74 | 0.06 | 13.19 | | 0.63 | 0.85 | 0.49 | 0.47 |
| 39 | 0.90 | 0.05 | 18.87 | | 0.81 | 0.99 | 0.63 | 0.60 |
| 40 | 0.72 | 0.05 | 14.12 | | 0.62 | 0.82 | 0.51 | 0.49 |

Note: all coefficients are significant at p < .001
