# Supplementary material for: Exchange and Communal Orientations (ECO) scale: The construction and validation of a method to measure target-specific relational orientations
Source: PLoS One. 2025 Jun 3;20(6):e0325232. doi: 10.1371/journal.pone.0325232 (PMC12132953; doi:10.1371/journal.pone.0325232)
Supplement: S2 Table — (DOCX) [file pone.0325232.s004.docx]

**S3 Table. Unstandardized and standardized factor loadings and discrimination indices for the second model CFA (final item pool; Study 1)**

| Item label | b | se | Z | 95CI lower limit | 95CI upper limit | β |
| --- | --- | --- | --- | --- | --- | --- |
| Exchange orientation – close person | | | | | | |
| Close2 | 1.00 | 0.00 |  | 1.00 | 1.00 | .77 |
| Close4 | 1.03 | 0.05 | 18.92 | 0.92 | 1.13 | .80 |
| Close5 | 0.93 | 0.05 | 18.65 | 0.83 | 1.03 | .72 |
| Close6 | 0.86 | 0.05 | 16.75 | 0.76 | 0.96 | .79 |
| Close7 | 0.99 | 0.06 | 17.11 | 0.88 | 1.10 | .75 |
| Close10 | 0.92 | 0.05 | 19.97 | 0.83 | 1.02 | .75 |
| Close12 | 0.95 | 0.06 | 15.61 | 0.83 | 1.07 | .72 |
| Close14 | 0.92 | 0.05 | 19.63 | 0.83 | 1.01 | .79 |
| Close17 | 1.05 | 0.05 | 20.63 | 0.95 | 1.15 | .79 |
| Close19 | 0.88 | 0.05 | 17.52 | 0.78 | 0.98 | .77 |
| Communal orientation – close person | | | | | | |
| Close21 | 1.00 | 0.00 |  | 1.00 | 1.00 | .73 |
| Close22 | 1.01 | 0.04 | 24.39 | 0.93 | 1.10 | .73 |
| Close23 | 0.92 | 0.06 | 16.71 | 0.81 | 1.03 | .63 |
| Close25 | 1.07 | 0.05 | 19.95 | 0.96 | 1.17 | .70 |
| Close26 | 0.90 | 0.06 | 16.21 | 0.79 | 1.01 | .58 |
| Close28 | 0.91 | 0.06 | 15.62 | 0.79 | 1.02 | .60 |
| Close30 | 0.92 | 0.05 | 17.69 | 0.82 | 1.02 | .64 |
| Close33 | 0.89 | 0.06 | 15.72 | 0.78 | 1.00 | .60 |
| Close35 | 0.99 | 0.05 | 18.18 | 0.88 | 1.09 | .67 |
| Close39 | 0.89 | 0.05 | 18.55 | 0.80 | 0.99 | .65 |
| Exchange orientation – acquaintance | | | | | | |
| Stranger2 | 1.00 | 0.00 |  | 1.00 | 1.00 | .66 |
| Stranger4 | 0.87 | 0.05 | 18.03 | 0.78 | 0.97 | .70 |
| Stranger5 | 1.07 | 0.05 | 22.74 | 0.98 | 1.17 | .74 |
| Stranger6 | 0.81 | 0.05 | 17.14 | 0.72 | 0.90 | .68 |
| Stranger7 | 0.84 | 0.05 | 18.35 | 0.75 | 0.93 | .67 |
| Stranger10 | 0.80 | 0.05 | 16.21 | 0.71 | 0.90 | .65 |
| Stranger12 | 0.83 | 0.05 | 18.08 | 0.74 | 0.92 | .67 |
| Stranger14 | 0.87 | 0.04 | 20.10 | 0.78 | 0.95 | .71 |
| Stranger17 | 0.88 | 0.05 | 17.34 | 0.78 | 0.98 | .66 |
| Stranger19 | 0.90 | 0.05 | 18.69 | 0.81 | 0.99 | .66 |
| Communal orientation – acquaintance | | | | | | |
| Stranger21 | 1.00 | 0.00 |  | 1.00 | 1.00 | .68 |
| Stranger22 | 1.02 | 0.04 | 24.00 | 0.94 | 1.10 | .67 |
| Stranger23 | 0.82 | 0.05 | 15.66 | 0.72 | 0.93 | .62 |
| Stranger25 | 0.95 | 0.05 | 19.59 | 0.85 | 1.04 | .68 |
| Stranger26 | 0.86 | 0.05 | 16.89 | 0.76 | 0.96 | .62 |
| Stranger28 | 0.79 | 0.05 | 15.38 | 0.69 | 0.90 | .61 |
| Stranger30 | 0.95 | 0.05 | 17.62 | 0.85 | 1.06 | .62 |
| Stranger33 | 0.77 | 0.05 | 15.01 | 0.67 | 0.87 | .61 |
| Stranger35 | 1.02 | 0.05 | 19.62 | 0.92 | 1.12 | .71 |
| Stranger39 | 0.91 | 0.05 | 19.20 | 0.82 | 1.00 | .63 |
